# Fragment-Based Immune Cell Engager Antibodies in Treatment of Cancer, Infectious and Autoimmune Diseases: Lessons and Insights from Clinical and Translational Studies

**DOI:** 10.3390/antib14030052

**Published:** 2025-06-24

**Authors:** Ge Yang, Mohammad Massumi

**Affiliations:** Department of Pharmaceutics, Rutgers University, Piscataway, NJ 08854, USA; gy118@scarletmail.rutgers.edu

**Keywords:** fragment-based antibody, immune cell engagers, T cell engagers, NK cell engagers, myeloid cell engagers, B cell engagers, immune checkpoint, antibody engineering, antibody translational medicine, antibody clinical development

## Abstract

Since the advent of recombinant DNA technologies and leading up to the clinical approval of T cell engager blinatumomab, the modular design of therapeutic antibodies has enabled the fusion of antibody fragments with proteins of various functionalities. This has resulted in an expansive array of possible mechanisms of action and has given birth to fragment-based antibodies (fbAbs) with immune cell engager modalities. In searchable databases, the preclinical development of these antibodies has shown promise; however, clinical outcomes and restructuring efforts involving these agents have produced mixed results and uncertainties. Amid budgetary cuts in both academia and industry, critical planning and evaluation of drug R&D would be more essential than ever before. While many reviews have provided outstanding summaries of preclinical phase fbAbs and cataloged relevant clinical trials, to date, very few of the articles in searchable databases have comprehensively reviewed the details of clinical outcomes along with the underlying reasons or potential explanations for the success and failures of these fbAb drug products. To fill the gap, in this review, we seek to provide the readers with clinically driven insights, accompanied by translational and mechanistic studies, on the current landscape of fragment-based immune cell engager antibodies in treating cancer, infectious, and autoimmune diseases.

## 1. Introduction

Antibody is one of the most essential classes of drugs in the contemporary pharmaceutical field. Despite its success as a class of drug, research and development of antibodies can face challenges due to many factors: (1) lack of clinical efficacy, (2) excessive toxicity, (3) poor pharmacokinetic profile, and (4) high immunogenicity. During periods of budgetary surplus, these factors might be considered “tolerable” in clinical development and receive approval. However, during periods of economic downturn as well as budget cuts, more frequent termination of drug development could occur when the above-mentioned factors occur. Given the availability of literature documenting clinical results of successful and unsuccessful drug candidates, an understanding of these valuable cases could help to provide insights for designing and polishing the drug candidate to meet clinical and commercial needs.

With the advancement of recombinant technologies in synthetic biology, antibodies with countless structural varieties could be crafted out of blueprints on computer software. Fragment-based antibodies (fbAbs) are a relatively general engineering approach for formatting recombinant antibodies. Compared to other chimeric IgG formats (such as chimeric bispecifics) or IgG-like structures that retain the main framework of the IgG, fbAbs strive to utilize the lowest molecular weight possible to achieve the desired therapeutic functionality as needed, by minimally drawing upon the fragments of IgG, such as scFv or Fc, fused with other molecules of interest. Due to their versatility, fragment-based antibodies are frequently the blueprints for constructing immune cell engagers. fbAbs were investigated in cancer, infectious disease, and autoimmune diseases, with more than 60 designs and more than 50 clinical trials on record [1,2], highlighting the attention attracted by their flexibility to meet therapeutic needs. In contrast to conventional antibodies, which function by antigen binding/neutralization as well as mediating Fc-dependent mechanisms, immune cell engagers are capable of more selectively and efficiently leveraging immune cells to engage in therapeutic modalities. For instance, in cases of treating cancer and infectious diseases, immune cell engagers could leverage immune cells to kill target cells or pathogens of interest, thereby allowing subsequent generation of adaptive immunity from destroyed remains of cancer cells or pathogens. In the cases of autoimmune conditions, immune cell engagers can modulate the generation of autoreactive immune cells and antibodies, thereby mitigating or even curing the condition.

**Figure 1:**
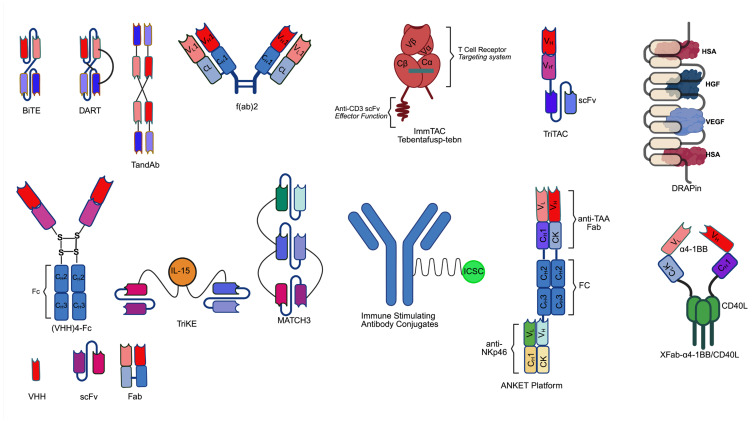
Structural diversity of fragment-based immune cell engagers discussed in this review.

A key advantage of the multidomain antibodies is their outstanding capability to be modularized, where each module (or domain) is assigned to bind an arbitrary target. With the aid of bioinformatics, an understanding of pharmacology gives rise to demands for the platform of tailorable targeted therapies. Besides targeting antigens to recruit immune cells and complement complex, designers of modular multivalent antibodies also have the liberty to simultaneously target multiple types of pathological antigens [3], in order to elicit synergistic effects [4], to target human serum albumin for extended half-life [5], to incorporate immune cell-modulating moieties to boost immune cell function [6], and to potentially neutralize target antigens like viral particles [7,8,9,10,11,12]. In terms of the mechanisms of action, those fbAb that entered clinical trials had these roles or mechanisms of action: (1) Immune cell engagement, (2) immune checkpoint modulation, (3) cytokine and chemokine modulation, (4) disease target binding for inhibition, activation, or neutralization, (5) payload toxicity [13]. Key parameters for engineering successful fbAb include: (1) persistence (pharmacokinetics), (2) affinity modulation [14], (3) conditional activation, (4) antigen selectivity, and (5) co-stimulation of the immune cell targets. The basic building blocks of these multivalent antibodies are usually derived from these two components: VHH (variable heavy domain of heavy-chain) and scFv (single-chain variable fragment). For those readers who are interested in more detailed comparisons between the structural activities of VHH and scFv, this review paper can be helpful [15]. In addition, protein subunits, such as Fc (fragment crystallizable) [16], PD-1 (programmed cell death protein 1), CD40L, cytokines, and immunostimulatory small molecules, are also embedded into the structure of the antibody to achieve enhanced pharmacokinetic and/or pharmacological properties (Figure 1).

## 2. Harness the Incidence of Anti-Drug Antibody and Pharmacokinetics

### 2.1. Mechanism for the Incidence of Anti-Drug Antibody (ADA)

The immunogenicity of antibodies poses a significant threat to the success of the treatment, as it introduces the binding of anti-drug antibodies (ADA) towards the antibody drug of interest. While pre-existing antibodies exist in the patients due to prior treatment or patient-specific immune response, treatment-emergent ADA is induced due to the treatment with the antibody. When the antibody is required for repeated dosing, the presence of ADA can cause the antibody drug of interest to be rapidly neutralized or cleared from the body, thus jeopardizing efficacy.

Immunogenicity of therapeutic antibodies in general depends on several factors. From the side of the patient, it could be due to genetic background (e.g., HLA (human leukocyte antigen) polymorphisms), and baseline immunogenic status (medication and disease condition). The product itself, its amino acid sequence, mechanism of action, dosing route, and manufacturing process can contribute to the incidence of ADA [17,18,19]. ADA could also induce various types of adverse effects, including but not limited to: type I hypersensitivity reactions and anaphylaxis (IgE ADA-mediated), type III hypersensitivity (inflammation caused by immune complexes, which can lead to fever, rash, hematuria, proteinuria) [20], alongside with reduced treatment efficacy and an increased potential risk of reaction against a similar class of drugs.

The amino acid sequence is the primary mechanism leading to the generation of ADA. Upon internalization by antigen-presenting cells (APCs, such as dendritic cells), the antibody is digested into peptides, being displayed to helper T cells via APC by MHC-II (major histocompatibility complex)-TCR (T cell receptor) interactions on the APC surface. Subsequently, the activated T cell, capable of identifying the peptide sequence of the antibody, can activate and maturate B cells with complementary BCR (B cell receptor), which are responsible for producing ADAs. In cases of T cell-independent pathways, the antibody can directly crosslink with the BCR, stimulating the differentiation of B cells into plasma cells to produce ADAs [18].

The extent of humanization is also a factor that is frequently being discussed. Yet, it may not serve as a definitive contributing factor for immunogenicity. According to two systematic/meta-analysis [21,22], the incidence of ADA for fully human antibodies ranges from 1.12% to 7.61% (1.12% for tezepelumab, 3.8% for golimumab, 7.5% for adalimumab, 7.61% for dupilumab), whereas the incidence of ADA for humanized antibodies ranges from 0 to 28%: (0% for omalizumab, 4.39% for reslizumab, 3.63% for mepolizumab, 8.35% for benralizumab, 10.9% for certolizumab, 16.0% for natalizumab and 28.0% for infliximab), indicating an overlap of ADA rates with or without humanization. Therefore, a fully human antibody may not always have a lower ADA incidence rate than humanized antibodies, and vice versa.

The mechanism of action of the antibody itself may also facilitate the generation of ADAs. Multivalent antibodies, capable of crosslinking more than one of the same antigens, may have higher chances of forming immune complexes, especially when the antigen is soluble and/or contains more than one epitope. Immune complexes, a gigantic scaffold of a crosslinked matrix of the antibody and the antigen, can be more easily captured and digested by APCs, serving as reservoirs for generating more ADAs. For instance, the anti-TNF (tumor necrosis factor) antibody has an ADA incidence rate ranging from 28% in dinutuximab to 66.7% in infliximab [18]. Aglycosylation for silencing the Fc is another main contributor to ADA, which can cause aggregation of the antibody in around 30–48% of the patients [19,23]. Anti-B cell antibodies, such as blinatumomab (≤2%), glofitamab (0%), and teclistamab (0–0.46%), could have lower ADA incidence rates [18], due to their mechanism of action to eliminate B cells that serve as the source for producing ADA.

Last but not least, the dosing route is an important factor in influencing the incidence of ADA. Subcutaneous (SC) route forces the antibody (since molecules > 20 kDa are too bulky to be able to directly enter capillary circulation) to traffic through interstitial matrix and reach peripheral lymphatic tissue, where the antibody is phagocytosed by antigen-presenting cells (APCs), and the immunogenic epitopes of the antibody may be presented to T cells, potentially eliciting an eventual ADA response [17], especially when the drug has to be dosed more frequently due to PK issues.

For instance, in the phase, I study of the BiTE (bispecific T cell engager) AMG212 [24], 96.7% (n = 31) of SC-dosed patients developed ADA (93.3% being neutralizing), whereas none (n = 16) of the patients dosed with continuous intravenous infusion (CIV) developed ADA. Those emergent ADA were characterized by early onset (cycle 2 day 1), resulting in increased drug clearance levels, contributing to rebound PSA levels, and reducing drug efficacy. Topically applied glucocorticoid cream, which could theoretically inhibit dendritic cell-mediated ADA induction, could not prevent or mitigate ADA development. In a more detailed analysis of peptide epitopes, it was found that three peptide fragments from the epitope of AMG212 were immunogenic to CD4^+^ T cells in a clinical memory recall assay to PBMCs (peripheral blood mononuclear cells) from patients who had been treated with AMG160 (which has 98.4% sequence homology with AMG212), as evidenced by elevated interferon-gamma levels. These observations also raise concerns for patients who need to be treated with analogous drugs. On the other hand, antibodies dosed via intravenous route can still possess immunogenicity, as seen in the case of BiTE AMG160 with a 20% rate of incidence for ADA [17].

### 2.2. Pharmacokinetic Challenges and Solutions

When an fbAb enters the human body, it can be consumed through the following ways: (1) renal filtration (especially for protein less than 70 kDa), (2) liver absorption, (3) target or receptor-mediated endocytosis, and (4) biochemical degradation [16]. The total number of half-life extension drugs published by the US Food and Drug Administration (FDA) had reached more than 50 by the year 2024 [16], highlighting the significance of these strategies. Clinically speaking, fbAb with a very low half-life and no half-life extending strategies may need frequent dosing and even extended periods of infusion, such as Blinatumomab, which may place a burden on the patient. Last but not least, the presence or availability of immune cells at the site of treatment is as important as the availability of immune cell engagers at the site of treatment. Physiologically-based pharmacokinetic (PBPK) model has been developed and suggested that the conditions as well as availability of the immune cells in the microenvironment can affect the treatment outcome of solid tumors [25,26]. With similar mechanisms of action, depleting tissue-resident B cells in autoimmune disease as well as pathogen-infected tissue-resident cells by immune cell engager should follow similar roles.

#### 2.2.1. Half-Life: Mostly Pro but Sometimes Con

Extended half-life can provide desirable clinical advantage to not only enhance the efficacy of the drug but also to enhance patient compliance and eventually the marketability of the drug. A trend in oncology has shown the emergence of half-life extended fragment-based immune cell engagers, which is to address issues as seen in the first-generation fragment-based antibody drug, blinatumomab. The short half-life of fbAb like blinatumomab gives rise to its continuous dosing regimen to make up for its rapid clearance, whereby up to nine cycles and over a hundred days of treatment duration may be needed [27,28]. Like yin and yang, despite the dosing regimen being less convenient, it possesses the safety feature of allowing rapid stop of treatment, which is in stark contrast with CAR-T, as CAR-T cannot be easily turned off in short time even with engineered off-switch designs that may need more than 12 h to days for complete “shutdown” [29,30,31]. Moreover, in the case of treating autoimmune disease through depleting B cells, transient depletion of pathogenic B cells would suffice, whereas a prolonged treatment regimen may not be needed nor even desired, as could be seen in the clinical study utilizing blinatumomab to treat refractory rheumatoid arthritis, where two doses of blinatumomab could suffice [32]. For the case of treating infection, treatment of chronic infection may prefer immune cell engagers with a longer half-life, whereas for treating acute infection, infusion could be preferable due to its ability to stop dosing in case of severe adverse events.

#### 2.2.2. Strategy to Improve Half-Life: Fc Fusion

To achieve a balance between permeability and retention in tissues, besides altering the sizes of the antibodies, one could opt to append an Fc domain, which can significantly extend the tumor uptake rate of the fbAb. Fc can bind to FcRn, which stands for neonatal Fc receptor. The binding occurs in a pH-dependent manner, allowing the antibody tagged with Fc to be taken up by cells into acidic endosomes. In the process, the antibody is protected from lysosomal degradation and is recycled and released back into the bloodstream [33]. For instance, DART (dual-affinity re-targeting) format antibody has structures similar to those of BiTE and a similar pharmacokinetic profile. By fusing DART to an Fc, the half-life of DART-Fc could reach as long as 26.6–45.8 h as could be seen in the case of PF-06671008 [34,35], or even better, up to 11–12 days as seen in the case of MGD013 and MGD019 [36,37,38,39]. Similarly, the Fc-fusion version of TandAb (tandem diabody) AFM24 (CD16A × CD123) is in clinical trials [40]. The half-life of TandAb ranges from 18.4 to 22.9 h for AFM11 [41,42] and 20.6 h for AFM13 [43]

Nevertheless, wild-type Fc possesses the capacity to interact with Fcγ, which can activate immune cells and complement pathways [44]. Such activation could be unwanted, especially when the antibody has a secondary immune cell-engaging subdomain, and in such case, the antibody could cause an Fc-mediated attack on immune cells recruited by the secondary immune cell-engaging subdomain. Therefore, researchers have engineered various Fc variants to inactivate their Fcγ-binding capacity while retaining the FcRn binding capacity. Examples of such engineered or mutated Fc include: L234A/L235A (also known as LALA), L234A/L235A/P329G (LALA-PG), as well as endosomal pH-depending binding variants, such as M252Y/S254T/T256E (YTE) or Q311R/M428L/N434W (REW) [16].

#### 2.2.3. Strategy to Improve Half-Life: Targeting Serum Albumin

For enhancement of circulation time in the bloodstream, attaching a serum albumin-binding domain to the antibody design can draw on the presence of serum albumin, analogous to a hitchhiking effect, which increases the bulkiness of the antibody and thereby slows down renal clearance. More importantly, similar to Fc, albumin can also bind to FcRn, allowing albumin-tagged antibodies to be recycled [45]. Indeed, anti-albumin design has been incorporated into the design of clinical phase fragment-based antibodies, such as TriTAC (Tri-specific T-cell activating construct) format antibody HPN328, which has a half-life of 71 h [46] that is significantly longer than that of BiTE molecules (~2 h), with similar molecular weight but without half-life extension [28].

## 3. Features of fbAb (Fragment-Based Antibody) Immune Cell Engager Platforms

BiTE (bispecific T-cell engager) platform, developed by Amgen, with the well-known example of blinatumomab, has been a hotspot for clinical development ever since the approval of blinatumomab by the FDA. From a structural standpoint, BiTE relies solely on a flexible peptide linker (GGGGS) between its anti-CD19 and anti-CD3 scFvs, which provides a minimalist structural requirement for bispecificity BiTE, such as blinatumomab, is produced in Chinese hamster ovary (CHO) cells, which secretes blinatumomab into the cell culture medium [47]. While the low molecular weight (MW) nature of BiTE allows it to bridge T cells and target cells in a distance mimicking natural immune synapse, low MW also contributes to significantly lower half-life. Therefore, unless half-life-extending engineering is performed, constant infusion or subcutaneous dosing route is needed to maintain therapeutic level concentration [47].

DART (dual affinity re-targeting) platform, developed by MacroGenics, with the example of CD3 × CD123 Flotetuzumab, incorporates an interchain disulfide bond at the C-terminus of its two scFvs, a feature absent in blinatumomab’s BiTE structure [48,49]. This covalent linkage stabilizes the heterodimeric configuration, preventing dissociation of the CD123 and CD3-binding domains under physiological conditions. The DART platform, due to the necessity of VH-VL pairing, is believed to reduce aggregation and enhance stability [48]. DART could be produced in CHO cells [47]. Similar to BiTE, due to its low MW nature, DART can also be fused to Fc to achieve substantial improvement for half-life.

ImmTAC (immune-mobilizing monoclonal T-cell receptor against cancer) platform, developed by Immunocore, is composed of an engineered TCR that can recognize MHC-peptide complexes and is linked via a peptide linker to an anti-CD3 domain (made of scFv in tebentafusp). Tebentafusp can be produced from E. coli [50], although refolding was needed [51]. A notable example is the FDA-approved tebentafusp (CD3 × MHC-gp100) [52]. The modality of the antibody is to mimic the recognition of MHC-peptide complex by TCR, followed by activation of the CD3 complex. Since the natural binding affinity between the TCR of cytotoxic CD8^+^ T cell for self-antigen-presented MHC is low due to the self-tolerance mechanism, the design of ImmTAC enables the possibility to target tumor-associated antigens using an artificial TCR [52]. In addition, ImmTAV (immune-mobilizing monoclonal T-cell receptor against the virus), a similar fbAb product that targets viral antigens instead, is also being developed to treat HIV [53]. Although the availability of the antigenic peptide may pose a challenge to developing drugs within the platform, the platform of ImmTAC and ImmTAV could potentially be used to target intracellular antigens displayed by MHC complexes, allowing uprooting of pathogenic lacking cell surface markers that are inaccessible to other classes of immune cell engagers or antibodies.

Fab derivatives are composed of two fab fragments or from fusing Fab and other proteins of interest. F(ab’)2, composed of two Fabs linked to an immunoglobin hinge region [52], has been gaining interest as an antivenom (e.g., for Black Widow spider venom) [54], antiviral (anti-COVID-19 [55,56]), antibacterial [57], antitumor drug [58]. Preexisting ADA could be a concern for F(ab’)2 due to its exposed hinge region as well as its equine origin, as seen in cynomolgus monkey [59]. Due to this feature, F(ab’)2 and its derivatives are being humanized. IMB071703 is an F(ab’)2 derivative, equipped with humanized Fab to target 4-1BB on T cell, fused to a trimeric CD40L protein cluster in order to mimic the natural condition of trimeric CD40L-CD40 interaction on B cells, eventually achieving attenuation of immune responses in autoimmune conditions. IB071703 was produced in CHO cells, though a V_L_-C_L_ kappa-CD40L_(n)_-C_H_1-V_H_ fashion of connection [60].

Fab fragments can also be combined with other antibody fragments, as can be seen in the case of TriNKET (trispecific NK cell engager therapeutics; Dragonfly therapeutics and Merck) and ANKET (antibody-based NK cell engager therapeutics; Innate Pharma). TriNKET is a class of tri and tetra-specific fbab, with scFv, Fab, and Fc components, targeting CD16, NKp46, cancer antigen, and/or potentially the beta chain of IL-2 (interleukin-2) receptor [61,62]. For instance, the TriNKET format antibody, DF1001 (CD16, NKp46 (natural killer cell p46-related protein), HER2 (Human Epidermal Growth Factor Receptor 2)), has shown efficacy in relapsed/refractory solid tumors [63]. Such trispecific design offers a mean to tailor different modalities to engage with immune cells. Similar to TriNKET, the ANKET platform antibody has two generations. The first-generation antibody targets CD16, NKp46, and a tumor-associated antigen (TSA), with the example of SAR443579/IPH6101, whose preliminary clinical results have been announced by Innate Pharma with multiple cases of complete remission [64]. The next generation of the antibody is tetravalent, with the addition of Fc for half-life extension [65]. Expression of ANKET is achieved in Expi-293F cells [65].

TriTAC (tri-specific T cell activating construct) platform, developed by Harpoon therapeutics (acquired by Merck/MSD), is a type of trispecific T-cell engager antibody, tandemly connecting humanized scFv/VHH that binds to a pathogenic antigen, CD3, and human serum albumin, effectively achieving T cell engagement of the pathogen as well as half-life extension [66]. While the fundamental structure of first-generation TriTAC may not exhibit significant difference compared to other half-life-extended T cell engagers, next-generation TriTACs, such as ProTriTAC with masked antigen-binding region, whose activation entails metalloproteinases in tumor microenvironment (allowing tumor-specific targeting [67], and TriTAC-XR, an extended-release version of TriTAC, whose activation relies on proteases (e.g., serum protease) available in the whole body potentially allowing a balance between therapeutic concentration and toxicity threshold in systemic circulation [68].

TandAb (tandem diabody), developed by Affimed, is a bispecific or tetraspecific antibody format with tandemly linked scFvs, possessing formats linked by two or four scFvs, in a manner where two copies of the same peptide chain are reversely overlaid and reinforced with disulfide bonds [42]. TandAb also serves as part of the ROCK platform (redirected optimized cell killing), with clinical examples including AFM13 (acimtamig), and AFM28 [40]. TandAb possesses building blocks similar to that of BiTE and DART format antibodies, its tetravalent design allows it to achieve enhanced activating effects [42]. TandAb can be expressed in mammalian cells, such as HEK293 cells [42]. Fc fusion, as seen in the case of clinical phase TandAb AFM28, has been engineered to extend the half-life of the antibody.

DARPin (designed ankyrin repeat protein) is based on naturally occurring ankyrin repeat domains, with roughly a total of 404 types of ankyrin repeats coded in human genes, which are typically the building blocks of human proteins in various functions [69]. Units of DARPin proteins appear to be thermodynamically stable, while its expression level could be a gram per liter level in E coli. expression system [69]. Clinical development of DARPin is ongoing. For example, the tetradomain DARPin, MP0250, contains five repeats, with domains recognizing human serum albumin, VEGF, and HGF [69]. DARPin platform includes AMG 506 (MP0310) [70], MP0274 [71]. Due to its ability to bind to serum albumin, DARPin format antibody could take advantage of the FcRn pathway, and it could achieve convenient dosing intervals of every 2, 3, or even 4 weeks [72,73].

TriKE (tri-specific killer cell engager) is a class of trispecific fbab, targeting NK cell antigen CD16, cancer/viral antigen, and includes tandemly linked scFv or VHH with an inserted IL-15 (interleukin-15) domain to stimulate NK cells [74,75,76]. While the two antigen-binding domains bridge the NK cell and the target cell, the IL-15 domain promotes the survival and persistency of NK cells. The TriKE CD16 × IL-15 × CD33 GTB-3550 has shown promising clinical results [77,78]. Future clinical development of the antibody will involve the replacement of the antibody component with VHH [79], which may reduce steric hindrance of IL-15 compared to first-generation TriKE design as well as circumvent the risk of aggregation in the scFv region [75].

The (VHH)_n_-Fc format antibody links VHH and Fc domains altogether, mimicking the Y-shaped IgG format, with the exception that one arm of the antibody can host multiple tandemly linked VHH, providing multiple options in designing the antibody. While tandemly liked VHH can provide maximum utilization of the molecular weight: functionality ratio, the overall size of the antibody will be small, since the average size of VHH is around only 15 kDa (in comparison, that of scFv is 25–30 kDa). Therefore, a multi-VHH design alone may require a half-life extension method, such as incorporation of Fc or anti-albumin domain. At present, erfonrilimab (KN046) is the most clinically advanced example of fragment-based immune cell engager incorporating multiple VHHs.

ISAC (immune-stimulating antibody conjugate) is a type of immune cell-engaging antibody, that utilizes conjugation of immune cell receptor ligands to antibody fragment to achieve immune cell engagement. ISAC relies on antibody or antibody fragment modules to target pathogenic antigens of interest and utilizes small molecules (oftentimes agonists of toll-like receptor, TLR) that are synthetically and covalently conjugated to the antibody of interest and can elicit anti-infection-like responses by antigen-presenting cells (APCs) [80].

## 4. T Cell Engagers (TCE)

T cell engagers (TCEs) are a class of drugs that bind to activating receptors on T cells, such as CD3. Since CD3^+^ T cells play major roles in immune memory (in addition to B cells), they have become a focus in the field of targeted immunotherapy. Because of the complexity of T cell immunology, their roles in cancer, infection, and autoimmunity are very different. In terms of the FDA-approved fbAbs (fragment-based antibodies), there are examples of blinatumomab, abciximab, ranibizumab, certolizumab pegol, emicizumab, caplacizumab, tebentafusp [81]. Among these drugs, blinatumomab and tebentafusp are used to treat cancer. In this section, the roles of T cells in these diseases will be examined based on the disease type.

TCE works by crosslinking T cells and the target cells, facilitating the formation of immune synapses, a process involving the rearrangement of cellular surface proteins that can bridge the T cell and the target cell [82]. The target cells presenting antigens of interest could be “healthy cells” that are responsible for autoimmune disease, cells infected with intracellular pathogens, and cancer cells. By binding to T cell activating receptors (such as CD3) and the antigen/target on the target cell, TCE can transiently connect T cell and the target cell, forming a cytolytic immune synapse. The signal from TCE binding then causes the T cell to release pore-forming protein perforin, which disrupts the membrane of the target cell, and apoptosis-inducer, granzyme, to activate cell death [83,84]. Moreover, the formation of the synapse can also promote the release of pro-inflammatory cytokines (IFN-γ and TNF-α) and the proliferation of T-cells [82]. Essentially, all cytotoxic T-cells can be engaged by this mechanism of action, including CD8^+^ T cells, CD4^+^ T cells, gamma-delta T cells, and NKT cells [83].

Compared to IgG and IgG-like TCE antibodies with larger sizes, fbAbs with smaller sizes, are capable of creating tight junctions (close distance binding or membrane proximity) between the T cell and the target cell, thereby creating an artificial immune synapse analogous to the ones observed during binding of antigen peptide-loaded HLA complexes and T cell receptor (TCR) [85,86]. Therefore, by exploring the size advantage of fbAb, TCE could have promising applications as preferable structural blueprints for designing TCE.

It is widely known that TCE and CAR-T are usually compared and contrasted with each other, specifically when it comes to CD19-targeted therapies (applicable for cancers and autoimmune diseases), where arguments have been made favoring CAR-T or [87] TCE (BiTE, bispecific T cell engager) [88], over the other. The favoring view [88] suggests the following: off-the-shelf (availability), lower cost, almost no manufacturability issues (comparatively), no need for lymphodepletion, significantly lower incidence of ≥grade 3 adverse effects in many categories, around one-fifth the cost of CAR-T, less risk of antigen loss, flexibility in targeting multiple antigens (e.g., as cocktails), and easier approaches to contain T cell exhaustion. The opposing view [87] claims that compared to blinatumomab, CAR-T has a higher complete response rate (CR) in adults (blinatumomab = 36–44%; CAR-T = 67–100%), superior trafficking to treat disease sites in CNS and extramedullary region, and better performance under higher tumor burden.

While the purpose of this review does not include a comparison between CAR-T and TCE, the shortcomings of TCE will be discussed, so as to shed light on its improvement. One key difference between blinatumomab and CAR-T is that both FDA-approved CAR-Ts (Tisagenlecleucel and Axicabtagene ciloleucel), are second-generation CAR-T products that are equipped with both CD3 and a costimulatory domain, namely either CD28 or 4-1BB. The presence of a costimulatory domain in CAR-T may put CAR-T at an advantage over blinatumomab by itself, since blinatumomab is equivalent of first-generation CAR-T, which does not draw on a co-stimulation signal. By efficacy, the first-generation CAR-T (with only a CD3ζ domain for stimulation), achieved much less efficacy than blinatumomab [89]. Moreover, a recent phase 1b trial [90] has shown that BiTE is able to achieve 92.3% (n = 13) of CR/CRh in oncology, if administered through the subcutaneous route, highlighting the potential to enhance the efficacy of fbAb TCE through improving dosing regimen. In alignment with this philosophy, TCEs with T cell costimulatory modality are being investigated in clinical trials, such as GNC-035 (ROR1 × CD3 × PD-L1 × 4-1BB) [91] and GNC-038 (CD19 × CD3 × 4-1BB × PD-L1) [92].

One key advantage of TCE is its capacity to benefit patients who have undergone CAR-T therapy or even antibody therapy targeting the same antigen. For instance, in treating relapsed or refractory large B cell lymphoma in a phase I/II trial [93], Epcoritamab, an CD3 × CD20, TCE, has demonstrated higher efficacy for patients who had relapsed from anti-CD20 therapy (but lower efficacy from disease refractory to anti-CD20). Similarly, those patients who are refractory to CAR-T would benefit less [93]. This trial represents the synergistic potential for combining conventional IgG, fbAb TCE, and CAR-T in treating cancer, which can provide a broader coverage of the tumor-associated antigen and avoid antigen escape. For instance, there are pediatric regimens which combine mini-hyper-CVD (cyclophosphamide, vincristine, and dexamethasone) with inotuzumab ozogamicin (INO), rituximab, and blinatumomab, resulting in relapsed/refractory B-cell ALL, with an overall response rate of 75% [94]. The mini-hyper-CVD regimen with INO, rituximab, and blinatumomab in adult patients yielded an overall response rate of 80% [94].

Last but not least, TCE is beneficial for patients who have immunodeficiency, such AIDS, where a clinical case report [95] has shown that a patient with refractory multiple myeloma and HIV could benefit from BCMA × CD3 teclistamab, where no HIV rebound was observed. In contrast, HIV infection could render the patient ineligible for CAR-T therapy due to the fact that detection of HIV (PCR, ≥13.2 copies/mL), is a violation of GMP standard [95].

### 4.1. T Cell Engagers in Cancer

As published in numerous pieces of literatures, T cell engagers have gained popularity in recent years in oncology, as evidenced by the FDA approval of mosunetuzumab (CD3 × CD20), Glofitamab (CD3 × CD20), Epcoritamab (CD3 × CD20), Teclistamab (CD3 × BCMA (B cell maturation antigen)), Elranatamab (CD3 × BCMA), Talquetamab (CD3 × GPRC5D (G protein-coupled receptor, class C, group 5, member D)), Tarlatamab (CD3 × DLL3 (Delta-like Ligand 3)), Tebentafusp (CD3 × gp100-MHC), and last but not the least, blinatumomab (CD3 × CD19) [49,96]. The following section will discuss the clinical outcomes of fbAb in hematological malignancies and solid tumors, as well as some insights from their preclinical studies.

#### 4.1.1. fbAb TCE for Treating Hematological Malignancies

Blinatumomab is the most well-studied fbAb so far, where the pros and cons from the roadmap of developing the drug serve as the best beacon for clinical and preclinical studies on fbAbs, consisting of an scFv-scFv fusion. The drug is approved to treat CD19^+^ acute lymphoblastic leukemia (ALL), and Philadelphia chromosome-positive B-cell precursor ALL that has relapsed or is refractory to treatment, A meta-analysis [97] of 18 studies involving 1373 patients treated with blinatumomab has shown a complete remission (CR) rate of 54% (95%CI: 44–64%). In randomized controlled trials [98], the blinatumomab-treated group showed significantly better outcomes compared to the chemotherapy group, with a 12% improvement in OS (overall survival), and a 2.16-fold higher event-free survival rate.

Compared to the safety profile of CAR-T (in the same meta-analysis with 446 patients [99]), in terms of ≥grade 3 AEs, blinatumomab has lower neurological toxicity (18% vs. 7%), lower CRS (19% vs. 3%), and a similar level of neutropenia (38% vs. 31%). One of the reasons for its relatively lower CRS and neurological toxicity is its dosing regimen, which, if designed properly, can mitigate toxicity, improve T cell functionality, and ultimately enhance efficacy. According to the latest drug label revised in 2018, blinatumomab is administered as a continuous infusion over 4 weeks, with a dose escalation of 0.5–90 μg/m^2^/d, followed by a 14-day or 56-day treatment-free interval or TFI [27,28]. The dose escalation allows clinicians to monitor the patients’ reactions from the patient and to alter the dose or provide additional support as needed.

The treatment-free interval (TFI) is crucial for maintaining the efficacy of the drug, as it could give some breaks for the T cells to prevent exhaustion. Analysis of the T cells from patients who underwent continuous treatment with blinatumomab has shown a reduction in cytotoxicity and IFN-γ level [100]. The same study [100] has also shown that continuous exposure of T cells to a half-life extended CD3 T cell engager antibody, AMG 562, can induce expression of T cell exhaustion markers (PD-1^+^Tim-3^+^LAG-3^+^), a reduction in T cell proliferation, cytotoxicity, release of granzyme B and IL-2. Furthermore, the adoption of a treatment-free interval or dasatinib (which can turn off T cell activation signaling), can also enhance treatment efficacy in terms of T cell proliferation, cytotoxicity, granzyme releasing, and the ratio of PD-1^+^Tim-3^+^LAG-3^+^ T cells [100].

While the pharmacokinetic challenges of blinatumomab pose significant challenges to its clinical development, efforts have been made to address this issue. Duvortuxizumab (MGD011) is a DART-Fc format CD19 × CD3 antibody intended to treat B cell malignancies [101], where the Fc domain could take advantage of the FcRn (neonatal Fc receptor) pathway. While the clinical results have not been published, an announcement from its parental company, MacroGenics, indicates that while multiple objective responses were observed, a number of patients experienced treatment-related neurological toxicities, even though these toxicities are similar to those seen in other CD19-targeted T cell engagers. Due to commercial competition, the development of duvortuxizumab is no longer attractive, highlighting the increasing commercial demand for higher standards of efficacy and safety for developing similar targeted TCEs. Similarly, during a phase I trial of AFM11, a CD19 × CD3 TandAb [41], development was halted due to a lack of efficacy and a significantly high incidence of severe adverse effects, including dose-limiting toxicities. As a result, neurotoxicity and limited efficacy led to the termination of the drug’s clinical development [101].

Amgen, in addition to developing blinatumomab, has also been developing many other BiTE drugs for treating hematological malignancies. One key lesson learned from these examples is the CD3 × BCMA AMG420 (also known as BI836909) for Relapsed and/or Refractory Multiple Myeloma (RRMM). During its phase I trial [102], it achieved an ORR of 31%, and a response rate of 70% and 50% of MRD-negative complete response in the maximum tolerated dose (400 μg/day) group [102]. Serious AEs were observed in 48% of patients (n = 20), with the most prominent being infection (n = 14), and polyneuropathy (n = 2). While the efficacy of AMG420 is promising, it was surpassed by its analog drug, the FDA-approved CD3 × BCMA antibody elranatamab, which achieved an ORR of 63.6% and CR of 38.2% in the phase 1 trial (n = 55) [103]. Although elranatamab outperformed AMG 420 in terms of efficacy, AMG420 still achieved a 70% response rate at its maximum tolerable dose (400 μg/day; 4-week infusion/6-week cycle) [102]. In comparison, Elranatamab showed its best response (ORR = 83.3%) at a dose of 1000 μg/kg administered subcutaneously [103]. Moreover, both elranatamab and AMG 420 demonstrated dose-dependent efficacy profiles, highlighting the need for balancing pharmacokinetics with toxicity in the pursuit of high efficacy. This may explain why Amgen incorporated the Fc domain in their subsequent product designs. AMG 330, a CD3 × CD33 BiTE, achieved a fair ORR of 14.2% but a serious AE incidence rate of 66% [104]. Due to a lack of efficacy and toxicity, AMG330 clinical development has been terminated according to the NIH website (NCT04478695) [101].

Besides BiTE, Flotetuzumab (MGD006), a CD3 × CD123 DART (dual-affinity retargeting) fbAb for treating acute myeloid leukemia, had achieved a promising outcome with a complete response of 11.7% for all groups and 26.7% in the patient group at the recommended phase 2 dose (RP2D) in the phase I/II trial [105], while the AEs are mostly grade 1–2 and manageable with appropriate interventions [105].

A preclinical research paper on Flotetuzumab (MGD006) suggests that the drug can modestly upregulate the expression of PD-1, but the exhaustion marker, TIM-3, remained low, with concurrent presence of high T-cell cytotoxicity following prolonged exposure to MGD006 suggests that the antibody has a lower propensity to induce T-cell exhaustion [106]. Despite the promising efficacy, flotetuzumab has been discontinued from development. As explained by the CEO of MacroGenics to the investors [107], Fc-incorporation, along with a next generation of CD3 component in MGD024, the next generation CD123 × CD3 drug intended to replace MGD006, is able to extend half-life and significantly reduce cytokine release. MGD024 is currently under clinical investigation [108].

#### 4.1.2. fbAb TCE for Treating Solid Tumor

Tarlatamab (AMG757), a CD3 × DDL3 BiTE antibody, has been approved by the FDA for treating extensive stage small cell lung cancer (SCLC) and therefore serves as the first BiTE approved to treat solid tumors. In its phase II trial [109], an ORR rate of 40% and 32% were observed in the 10-mg and 100-mg groups, respectively. Such efficacy is promising, as limited options are available for pretreated small-cell lung cancer patients. Compared to blinatumomab, its predecessor BiTE molecule with a half-life of 2.1 h [110], tarlatamab, by fusing with an Fc domain [111,112], is able to achieve a clinical terminal elimination half-life of 5.7 days [113]. Therefore, while blinatumomab requires continuous infusion on a daily basis, tarlatamab only requires a one-hour infusion on a biweekly basis, significantly reducing the burden on patients. (DLL3 is a Notch ligand that) plays a role in neuroendocrine differentiation and SCLC (Small Cell Lung Cancer) tumorigenesis [111,112]. Tarlatamab is able to recognize cells with low levels of DLL3 expression with less than 1000 DLL3 molecules per cell, demonstrating efficacy against patient-derived xenografts and orthotopic mouse models [111,112]. Compared to blinatumomab, tarlatamab has a binding affinity of 3 nM towards CD3 and 3.1 nM towards DLL3, which is much higher than that of blinatumomab (260 nM towards CD3 [114]). As a result, likely due to the stronger binding and activation, PD-L1 expression was significantly upregulated after co-incubating T cells, cancer cells, and tarlatamab [111,112]. Such observations, also observed in the study of AMG562 [100], are potential signs of T cell exhaustion, supporting the rationale for treatment-free intervals or combination with immune checkpoint blockers (ICBs).

The BiTE platform has provided successful examples. Although some business reorganization and strategic decisions may contribute to the termination of BiTE development, from insights in business development [115], the BiTE platform from Amgen’s oncology devision stll has 4 active BiTE pipelines (excluding the two marketed drugs, blinatumomab and tarlatamab) and 14 inactivated BiTE molecules that were terminated after phase I. Except for portfolio prioritization, clinical and scientific reasons, including toxicity, need for infusion, and immunogenicity, were major reasons why these BiTEs were terminated [115].

Similar to TriTAC HPN424, the PSMA × CD3 BiTE pasotuxizumab (BAY 2010122/AMG212) was clinically tested to treat metastatic castration-resistant prostate cancer [116]. While some efficacies were noted (≥50% PSA decrement in 3 patients, n = 16; one patient showed complete regression of soft-tissue metastasis), 81% of patients have experienced at least one ≥ grade 3 AEs. BiTE AMG211 (MT111/MEDI-565), a CEA × CD3 BiTE [117], has shown high immunogenicity so that the therapeutic window could not be defined in a clinical trial, accompanied by insufficient exposure to establish an objective response [101]. BiTE AMG 596 (also known as etevritamab), an EGFRvIII (epidermal growth factor receptor variant III) × CD3 BiTE [118], has been used to treat EGFRvIII^+^ recurrent glioblastoma (RGBM). Serious adverse effects were observed in 50% of patients (n = 14), and 1 out of 8 patients had a partial response.

While the clinical landscape of BiTE has ups and downs, a similar trend could be observed in the TriTAC platform. Similar to BiTE tarlatamab, HPN328, a CD3 × DLL3 × albumin TriTAC fbAb, also achieved promising clinical efficacy, exhibiting antitumor activity against relapsed/refractory metastatic SCLC (confirmed ORR = 50%), neuroendocrine cancer (NEC, other than prostate neuroendocrine cancer; confirmed ORR = 44%), while achieving linear PK and a median half-life of 71 h [46]. Other TriTAC format antibodies, such as CD3 × PSMA × albumin HPN424 [119] and CD3 × mesothelin × albumin HPN536 (NCT03872206), are in clinical phases. HPN424 [119] has a well-tolerated safety profile, but disease progression (metastatic castration-resistant prostate cancer) has led to discontinuation of the treatment. PSA level declined in 21% of patients (n = 63) with follow-up at 24 weeks. Heavy pretreatment (<2 prior systematic therapies), and the presence of antigen-escape or DLL3-negative tumor subtype in prostate cancer patients [120], could also be contributing factors. Last but not the least, in preclinical studies, compared to other peer TriTACs, HPN424 [66] and HPN536 [121], which are configured in a tandem domain format of anti-CD3-anti-albumin-anti-tumor target configuration (also known as the C:A:T format), HPN328 is configured in an anti-tumor target-anti-albumin-anti-CD3 configuration (T:A:C format) [122]. It was noted by the authors [122] that the C:A:T format HPN328 is 3.1-fold more potent than its format in T:A:C in vitro, suggesting that geometry influences the binding between the anti-DLL3 domain and DLL3.

#### 4.1.3. Improving and Examining the Safety of TCE in Treating Solid Tumors

As mentioned in the previous section, incorporation of the Fc domain into the design of the antibody could facilitate its half-life. Nevertheless, if the incorporated Fc is unsilenced, it could potentially cause issues by unintentionally depleting immune cells of interest. An antibody with unsilenced Fc and anti-CD3 domain can crosslink Fc-effector cells and T-cells, leading to mutual attacks by the immune cells [123], as is evidenced by catumaxomab, which was withdrawn in 2017 [123]. As shown in preclinical studies [124,125], TCE with active Fc and anti-CD3 domain not only failed to direct T cells to the tumor but also induced T cell depletion and aggregation of T cells in the lung (for anti-HER2 TCE). Meanwhile, Fc-silenced TCEs do not sequester T cells in the lungs but are able to induce T cell infiltration in tumors. Mutations like N297A and K322A can reduce ADCC or CDC towards T cells, the incidence of CRS, while preserving the cytotoxicity of T cells and the consistency of pharmacokinetic profile [123,124,125].

One example is PF-06671008, a P- cadherin × CD3 DART with an extra Fc domain for an extended half-life of ~4.4 days [35]. PF-06671008 was tested in a phase I clinical trial (n = 27), and its further development was trial terminated due to lack of efficacy [34]. The clinical trial also showed that ≥grade 3 AEs (adverse events) occurred in 62.9% of patients, with an incidence of ≥grade 3 CRS at 18.5%, ≥grade 3 lymphopenia at 33.3%, and ≥grade 3 Hypophosphatemia at 18.5%. Pre-clinical research on PF-06671008 [35] suggests the antibody has good tolerability at even a dose of 10 μg/kg in hPBMC/hFcRn-humanized mice, indicating that preclinical mouse model may not detect immune cell depletion by Fc-mediated mechanisms without cellular characterization. The Fc domain of the PF-06671008 DART antibody includes only knob-in-hole and disulfide bond engineering [35], but lacks modifications that deactivate Fc gamma receptor binding [126,127], posing a potential threat to its efficacy.

When investigating the impact of off-tumor on-target toxicities using animal models, three approaches are generally employed. The first approach would be to use genetically modified animals expressing human genes encoding the target of interest, and the second approach would be to use non-genetically engineered animals with “animalized” version of the drug of interest (i.e., an antibody engineered to precisely target the animal homolog of the human protein target), and the third approach would be to test human drugs in non-human primates, like cynomolgus monkeys. Although all three approaches have their own advantages, their disadvantages are profound. The first approach may not recapitulate the interaction of the knock-in target protein with other proteins in the animal in a physiological context, the second approach may not be able to recreate a drug analog that targets the animal target with a sufficiently similar binding modality, and the third approach can overlook subtle differences between human and non-human primate.

The abovementioned first and third approaches were utilized in the development of solitomab (AMG110 or MT110), an EpCAM × CD3 BiTE. Development of the drug terminated, due to on-target dose-limiting toxicity seen in clinical trials. 95% of patients have ≥grade 3 treatment-related adverse effects, with major TRAEs observed in the lung, liver, and GI tract. Only one unconfirmed partial response was observed among sixty-five patients [112,128], yielding a maximum tolerable dose below the potentially therapeutic dose level. Dosing selection was made from 1 μg to 96 μg/day, and it was determined that the maximum tolerated dose (MTD) is 24 μg/day. IHC shows CD3^+^ T cell infiltration of EpCAM^+^ duodenal epithelium as well as damage to crypt structure with villus collapse by H&E staining [112,128].

The preclinical testing of solitomab highlights the limitations of animal models for the prediction of clinical toxicity for immune cell engagers and the necessity for more critical experimental design and data interpretation. When treating mice (Female BALB/c mice (Janvier, Le Genest-Saint-Isle, France) 7 weeks old) for two days with 50 μg/kg/day of muS110, the murine version of AMG110, weight loss, hypoactivity, and diarrhea were observed [128]. Similar to results in clinical biopsy, mouse necropsy shows significant damage to vacuolated enterocytes in duodenum tissue, accompanied by granzyme B-expressing CD4^+^ lymphocyte infiltration. Interestingly, in early preclinical study of muS110 BiTE [129], muS110 was well-tolerated up to 50 μg/kg, while efficacy could be achieved as low as 5 μg/kg, both of which concentrations are significantly higher than those seen in later in vivo study or clinical study (24 μg/day) [112,128]. No significant weight loss was observed at 12.5 μg/kg until reaching the 12.5–400 μg/kg range in a BALB/c CT-26 IV tail vein xenograft model. Moreover, PBMC-humanized mice did not seem to display toxicity as well [129]. It could be noted that the application of IHC could have been helpful in identifying the toxicity profile of the drug, but still, the MTD in mice and humans is drastically different.

Importantly, the difference of MTDs of solitomab in mouse study and human study could not be explained by the binding affinity (for EpCAM, K_D_ muS110 = 21 nM, K_D_ MT110 = 13 nM), (for CD3, K_D_ muS110 = 2.9 nM, K_D_ MT110 = 100 nM) [129]. Despite comparable anti-EpCAM affinity and lower anti-CD3 affinity in MT110, solitomab could even be administered to cynomolgus monkey at a high concentration for 1 mg/kg for a period of 6–10 days in the pharmacokinetic study [130], suggesting a potentially safer profile in cynomolgus monkey than in mice.

These preclinical findings underscore the limited translatability of preclinical antibody safety research to clinical settings in solid tumors, where the on-target off-tumor effect can frequently damage organs and tissues. Organ-on-chip models [131] could be an effective method to assay on-target off-tumor toxicity of immune cell engagers. It was seen that lung-chip and intestine-chip, as well as their organoids, could help to reproduce and help to predict target-dependent toxicity of immune cell engager. For instance, in a study investigating anti-FOLR1 (folate receptor-1) and anti-CEA (carcinoembryonic antigen) therapy, under the presence of PBMC, signs of on-target off-tumor toxicity were observed, as evidenced by elevated levels of IFN-γ, granzyme B, and IL-6 by PBMC, by anti-FOLR1 engager against healthy lung and kidney cells, and by anti-CEA antibody against healthy duodenum cells in the chip [131].

Another methodology for designing safer T-cell engager is by targeting tumor-specific antigens displayed by the major histocompatibility complex (MHC), through engineering a TCE that incorporates a T cell receptor (TCR) that can recognize the tumor antigen-MHC complex. At present, such antibody design has been achieved to target tumor-associated antigen, with arguably the best-known example being the FDA-approved drug tebentafusp (in the ImmTAC format), containing an anti-CD3 scFv and a TCR domain that recognizes the complex of MHC-I (HLA-A*02:01) and the proteosome-digested fragment of gp100 (glycoprotein 100). From a meta-analysis [132], it has a partial response rate of 8% and a stable disease rate of 36% in treating metastatic uveal melanoma, a rare form of solid tumor originating in the eye. Other therapies, such as the anti-PD-1/PD-L1 immune checkpoint blockers, have limited efficacy due to the lack of PD-L1 expression in the primary tumor and liver metastasis. In a propensity score-weighted analysis [133], the 1-year OS rates for tebentafusp and pembrolizumab were 73% and 59% in a randomized, phase III trial (IMCgp100-202; N = 378, respectively, while in the single-arm GEM1402 (N = 52)) in untreated metastatic uveal melanoma (mUM) the 1-year OS rate for nivolumab plus ipilimumab (N + I) in mUM was 52%. According to the phase 2 trial result of tebentafusp [134], among the 59% of patients with ≥grade 3 AEs, the breakdown of the most common AEs was as follows: rash (16%), hypotension (8%), CRS (4%), and pruritus (4%). Tebentafusp has an affinity of K_D_ = 38 nM towards CD3 [135] compared to that of blinatumomab [114]. Since the target of the antibody gp100, is abundantly presented on uveal melanoma cells, the resulting digested form of the peptide displayed by the MHC-complex gp100 has a decent amount of display on the cancer cells [136]. However, given that healthy melanocytic tissue also expresses gp100 [137], rashes caused by T cell and macrophage activation are also prominent adverse effects seen in clinical trials [133].

Tebentafusp not only serves as the harbinger for fbAb and TCE development in treating solid tumors but also paved the way for developing antibody therapies against MHC-peptide complexes as well as neoantigen therapy, a promising avenue for curing even the most recalcitrant types of cancer. Because MHC is able to display peptide fragments of intracellular origin, the antigen repository accessible to antibodies targeting MHC is more diverse than that of antibodies. Moreover, MHC-peptide display can present tumor-specific antigen (TSA), which can theoretically eliminate the risk of on-target off-tumor toxicity. Such methodology is promising in immuno-oncology. One of the most famous clinical studies [138] of neoantigen therapy involved a vaccine composed of up to 20 neoantigens plus atezolizumab, in treating pancreatic ductal adenocarcinoma (PDAC).

Among the 50% of patients who responded to the study, the recurrence-free survival was not reached at a median follow-up of 3.2 years and that of the non-responders was 11.0 months. In the study, the antigens were formulated as mRNA in lipoplex nanoparticles. Each of the mRNA encoded up to 10 MHC-I and MHC-II neoepitopes, and 11% of antigen epitopes were able to induce a T cell response sufficient to be detectable via ex vivo IFN-γ ELISpot. Besides neoantigen vaccines, engineered autologous T cell receptor (TCR) therapies recognizing specific MHC-peptide complexes have also shown promises, such as the FDA-approved Afamitresgene autoleucel, which can recognize the MAGE-A4 peptide presented by HLA-A*02 for treatment of advanced synovial sarcoma [139]. Despite the promising aspects of neoantigen vaccines and TCR cell therapy, the manufacture time and treatment cost are still major roadblocks due to the need for individualization, and these disadvantages can be addressed by off-the-shelf fbAbs like tebentafusp. Antibody and TCR-based fbAbs against MHC-peptide complex for mutant p53 (tumor protein 53) [140,141,142] or KRAS (Kirsten rat sarcoma viral oncogene homolog) [143,144], have been developed (see one review here for more details [145]), serving as arsenals for antibody engineers to generate optimized versions of fbAbs.

Similarly, besides tebentafusp, other formats of ImmTACs, like IMC-C103C (CD3 × MAGE-A4 (Melanoma-associated antigen A4)), and IMC-F106C (CD3 × PRAME (Preferentially Expressed Antigen in Melanoma)) [1], are also being tested in clinical trials. A clinical trial of IMC-F106C in cutaneous melanoma (CM) for patients who had been treated with an immune checkpoint inhibitor (ICI) has shown a combined partial response (PR) plus stable disease (SD) of 61%, with the most common AE being grade 1/2 CRS in 50% of patients [146]. On the other hand, despite showing promise in clinical trials, IMC-C103C has been discontinued from development by Roche, which does not seem due to lack of safety or efficacy but rather due to business reasons [147], likely due to potential competition from the newly FDA-approved anti-MAGE-A4 TCR Afamitresgene autoleucel [139].

#### 4.1.4. Targeting 4-1BB and CD28 in TCE Design

Targeting T cell costimulatory receptors, such as 4-1BB and CD28, by fbAb, has also been investigated for clinical efficacy. NM21-1480, an scMATCH3 (scFv3) format fbAb with tri-specificity for PD-L1, 4-1BB, and human serum albumin (HSA), has also been tested for clinical safety and efficacy and serves as a notable example for discussion. The antibody marks both an example of a trispecific fbAb, demonstrating successful half-life extension and controllable 4-1BB-medaited T cell activation [148,149]. The 4-1BB-targeting scFv of NM21-1480 binds to a membrane-distal epitope of 4-1BB. Since clustering of at least three 4-1BB molecules is required for stimulation of 4-1BB signaling [150,151], the activation of 4-1BB can be achieved once the antibody is bound to a target cell expressing PD-L1. The ultrahigh binding affinity of NM21-1480 towards PD-L1 allows the antibody to serve as an anchor on the cancer cell for T cells, transforming the cancer cell into a platform for 4-1BB clustering to enable tumor-specific activation. The lower affinity of NM21-1480 for 4-1BB prevents it from effectively clustering 4-1BB [148,149]. This design of NM21-1480 also suggests that multivalent antibodies targeting more than one 4-1BB domain may be less desirable compared to a monovalent approach for targeting 4-1BB. A preclinical study [148] shows that NM21-1480 can lead to tumor regression in 60–80% of animals across multiple cancer types, including non-small cell lung cancer (NSCLC), breast adenocarcinoma, and colorectal adenocarcinoma. The study also shows no detectable systematic cytokine release, confirming localized activity. A phase I trial (N = 26) [149] for unresectable solid tumors, has shown that NM21-1480 exhibits a disease control rate of 57% in the 24–800 mg dose range groups, where 62% of patients had received anti-PD-(L)1 therapy. The trial also shows that NM21-1480 has no maximum tolerated dose, with no incidence of grade > 3 AE, endorsing the safety of the fbAb platform. Moreover, the drug is dosed every two weeks [149], in alignment with its preclinical profile for a half-life of 14 days in cynomolgus monkeys [148], indicating a major improvement in pharmacokinetics and dosing regimen compared to the non-half-life-extended blinatumomab. Other similar 4-1BB targeted therapies are being developed, such as GEN1046 (4-1BB × PD-L1, [152]), and TJ-CD4B/ABL111 (4-1BB × Claudin 18.2, [153]).

Like targeting 4-1BB, targeting CD28 has also been investigated. CD28-targeted therapy, unlike CD3-targeted therapy, provides direct co-stimulation independent of TCR signaling, enabling activation even in environments with suboptimal antigen presentation [154]. CD28 bispecifics can also counteract T cell exhaustion by delivering strong co-stimulatory signals that restore metabolic competence in T cells, as evidenced by increased mitochondrial biogenesis and enhanced glycolytic capacity in treated cells [154].

### 4.2. fbAb TCE in Treating Infectious Disease

As will be discussed in this section, T cell engagers have been studied and clinically tested for viral infections [155,156,157,158,159], and the efficacy and toxicity of these agents have been examined. Activation of T-cells by TCE engager can usually lead to the release of a substantial amount of IFN-γ (interferon-gamma) and TNF (tumor necrosis factor). In combating viral infection, IFN-γ can downregulate the expression of viral entry receptors (e.g., claudin-1 for HCV (Hepatitis C Virus) and CD4 for HIV), inhibit RNA synthesis, block protein synthesis, induce RNA degradation for HBV, disrupt capsid assembly for HBV, and destabilize cccDNA (covalently closed circular DNA) for HBV (Hepatitis B Virus) [160]. IFN-γ can also upregulate MHC processing and presentation, which can facilitate the generation of adaptive immune responses [161,162]. TNF can induce RNA degradation (in SARS-CoV-2, EMCV (Encephalomyocarditis Virus), and influenza), help activate NK cells and M1 macrophages for pathogen clearance, and synergize with IFN-γ [163].

Moreover, TCE-activated T cells can release a substantial amount of cytokines contributing to CRS. For instance, COVID-19 infection and TCE activation are both characterized by elevated levels of IL-6, IL-10, TNF-α, and GM-CSF (granulocyte-macrophage colony-stimulating factor) [164,165,166,167,168,169]. While CRS can be helpful for combating infection, TCE and CAR-T therapies designed to treat COVID must account for the risk of CRS [166,167], which, when compounded with the CRS resulting from COVID infection [168,169], may cause severe adverse effects and even potentially death of a patient. For instance, an analysis has shown that B-cell depleting TCE can significantly increase the mortality of COVID patients compared to the canonical IgG-based B-cell depletion drug rituximab (57,1% vs. 12.5%, *p* = 0.002), yielding an adjusted odds ratio of 7.08 (95% CI = 1.29–38.76 [159]. Therefore, both the safety and efficacy of the antibody should be considered during the design process.

#### 4.2.1. fbAb TCE in Treating SARS-CoV-2 and Influenza (Pre-Clinical)

SARS-CoV2-infected cells display spike proteins on the cell surface. After SARS-CoV-2 viral infection, the viral spike protein is synthesized in the endoplasmic reticulum, and is substantially transported and integrated into the host cell membrane, serving as anchorage points for adhering to and infecting neighboring cells [170,171,172]. Considering this feature, two fbAbs with the CD3 × ACE2 (angiotensin-converting enzyme 2) and CD3 × spike protein [166,173] BiTE designs can achieve efficient outcomes in pre-clinical studies. Research [173] for S-BiTE has shown that the binding region of S-BiTE within the RBD is evolutionarily conserved across variants (including Omicron BA.1/BA.2/BA.4/BA.5). S-BiTE can also induce activation of IFN-γ and TNF when added to co-cultures of T cells and spike-expressing cells.

Influenza, while targetable by vaccinations, can still be potentially treated by T cell engagers. Recent research [174] has identified a broadly cross-reactive TCR from CD8^+^ T cells capable of targeting 9–12 influenza virus variants spanning from 1918 to 2024, where the targeted epitope is highly variable but remains immunodominant. Such epitopes can be used for screening in display platforms, while platforms such as ImmTAV [155,156,157,158], which utilizes TCR as part of its structure, can directly draw on the availability of the TCR to rapidly develop TCE.

#### 4.2.2. fbAb TCE in Treating HIV

A key challenge for HIV treatment lies in the presence of a latent viral reservoir, in which a population of CD4^+^ T cells harbors replication-competent provirus that persists despite antiretroviral therapy (ART) [175]. These cells (also known as viral “reservoirs”) can evade immune detection due to minimal viral protein expression, posing challenges for treating HIV with antibodies and T-cell-based therapies. Latency-reversing agents (LRAs), such as histone deacetylase (HDAC) inhibitors like vorinostat and romidepsin, have shown limited success in reactivating latent HIV and inducing re-expression of viral antigen [175]. Theoretically, polyclonal or multispecific antibodies could exploit multiple epitopes or different viral antigens, whereas ultrahigh-affinity antibodies may be able to overcome the scarcity of cell surface viral antigen density.

One of the best examples of the ultrahigh affinity approach is the ImmTAV fbAb platform, with the clinical stage examples of IMC-M113V and IMC-I109V. The platform utilizes anti-CD3 scFv as well as a picomolar-affinity TCR to bind to HLA (HLA-A*02:01) on HIV-infected cells that are displaying gag peptides [157,158]. Due to the ultrahigh-affinity nature of the fbAb, it is capable of eliminating HIV-infected CD4^+^ T cells from ART-treated patients, and the efficacy is correlated with HIV gag (Glycosaminoglycans or Group-specific antigen) expression [157]. The study also showed that immune dysfunction (PD-L1^+^TIM-3^+^, T-cell immunoglobulin, and mucin domain-containing molecule-3) in T cells from ART-treated patients was associated with reduced efficacy compared to T cells from healthy sources, suggesting a potential need to combine immune checkpoint inhibitors and/or interleukins for correcting patients’ T cells for maximum efficacy. In addition, in the phase 1/2 trial update from the company [158], IMC-M113V demonstrated good tolerability (no serious adverse events), dose-dependent cytokine increase, as well as dose-dependent viral control after interruption of antiretroviral treatment (ART). Three patients with evidence of viral control were suspended from ART, for analytical treatment interruption as specified in the protocol of the clinical trial for up to twelve weeks. The three patients experienced viral rebound, followed by viral reduction to approximately 200 c/mL, which occurs in less than 1% of all patients living with HIV in general [176], suggesting the presence of an immune response against the virus [158].

Besides ImmTAV, the DART-Fc format antibody, MGD014, which directly binds to gp120 of HIV (CD3 × gp120), was also clinically examined, showing good tolerability in a phase I trial [177]. Nevertheless, when used as standalone therapy, neither MGD014, nor the DART-Fc MGD020 (CD3 × gp41), were able to decrease persistent viral infection biomarkers, such as cell-associated viral RNA or time to rebound of viremia after ART interruption [177], highlighting the need to apply combinatory approach to enhance the efficacy.

Simultaneously reactivating latent HIV and targeting viral antigens has also been attempted, as evidenced by preclinical studies of a trispecific TCE targeting CD3/CD28 × gp120 [178,179]. The platform utilizes an antibody fragment that targets a conserved sequence in the CD4-binding region of gp120, allowing identification of viral antigen, whereas binding of CD3 could stimulate latent infected-T cells (ACH2, J1.1, and OM10) and cause reactivation of latent HIV. Overall, binding of gp120 and CD3/CD28 can cause latent T cells to be reactivated [180], expressing HIV and HIV-related proteins, forcing the reactivated HIV-infected T cell to be immunogenic for the attack by the gp120 × CD3 TCE, ultimately achieving both in vitro (HIV patient cells) and in vivo efficacy in rhesus macaques [178,179].

#### 4.2.3. fbAb TCE in Treating HBV

Immunotherapy for HBV faces challenges in curing HBV infection, such as difficulty in eliminating cccDNA reservoir, suppression of pathogen recognition receptors (PPPs), and the exhaustion of CD8^+^ T cells from chronic infection [181]. Unlike the usage of T cell-based therapies in treating HIV, controlled activation is crucial in treating HBV, since HBV can hijack healthy liver cells. T cells can cause hepatocyte apoptosis and liver damage, which can be exacerbated by the pre-existing inflammatory microenvironment [181]. Moreover, the clonal diversity of HBV should also be addressed to avoid mutant escape.

The MHC-restricted targeting approach could be helpful for limiting the toxicity of TCE. Similar to IMC-M113V, IMC-I109V [155,156] is also part of the ImmTAV variant fbAb utilizing a TCR-based method that can recognize a conserved region of HBV Env protein. In a clinical trial, IMC-I109V displayed good tolerability, with no reports of serious adverse effects, and no association with CRS as seen in preliminary clinical results. A rise of IL-6 and alanine aminotransferase levels was also observed, indicating a response consistent with the mechanism of action of the antibody [155,156]. Efficacy from the prospective phase 2 trial will need to be confirmed, as in the phase 1 trial, a decline in serum viral surface antigen was observed in a low-dose group, albeit with a transient response duration [155,156]. Moreover, a preclinical study has demonstrated that ImmTAV can also be used to target HBV Pol (DNA polymerase), Core (core protein), and Env (envelop protein), by activating T cells and promoting the release of inflammatory cytokines [182].

Similarly, preclinical studies of TCE fbAbs with various formats directly targeting CD3/CD28 × HBV antigens have been developed [183]. The study compared BiMAb (four scFv connected to an Fc-delta by a G4 or G4S3 linker) and FabMAb (Fab connected to an scFv with a G_4_S_3_ linker). Each BiMAb or FabMAb binds to only CD3 or CD28. It was observed that FabMAb and BiMAb performed similarly in activating CD8^+^ T cells (LAMP1^+^CD25^+^, Lysosomal-associated membrane protein 1) when equipped to bind to CD3, whereas when binding to CD28, only FabMAb exhibited activation but not BiMAb. Co-treatment with both CD3- and CD28-fbAb TCEs can achieve a synergistic effect in T cell activation. Interestingly, during prolonged co-culture of the fbAbs (mixture of CD3 and CD28-TCE) with PBMC and HBV-infected HepG2-NTCP cells, from day 10–12 in the medium, FabMAb achieved superior control of BHeAg, compared to BiMAb.

### 4.3. fbAb TCE in Autoimmune Disease

Efforts have been made to deplete B cells with the aim of resetting B cell immunity to eliminate the production of autoantibodies by B cells and activation of autoreactive T cells observed in many autoimmune diseases, such as systematic lupus erythematosus (SLE), rheumatoid arthritis (RA), and systematic sclerosis. Studies utilizing rituximab in B cell depletion therapy for treating SLE have shown a significant reduction in the levels of anti-dsDNA [184,185,186]. Nevertheless, rituximab cannot reduce the level of anti-histone, anti-RNP (ribonucleoprotein) autoantibodies [187]. It is believed that the long-term presence of high serological levels of anti-Sm (Smith antigen)/RNP and Ro/La autoantibodies is likely due to germinal center (GC)-derived long-lived plasma cells as well as memory B cells, whereas the transitory presence of anti-dsDNA autoantibodies is likely an indication of the presence of short-lived plasma cells [186]. Therefore, targeting both short-lived and long-lived plasma cells is needed to address the therapeutic need of resetting the B cell immunity. Since CD20 is a marker for less mature B cells while CD19 is a marker for more differentiated B cells [188], CD19 targeted therapies are believed to have more comprehensive coverage of B cell lineages that fall under different growth stages and therefore offer greater potential to cure autoimmune diseases. Indeed, CD19 profiling of SLE patients who had received CD19 CAR-T therapy after three months showed that autoantibodies against dsDNA, Ro/La, and histone were reduced in five out of six patients [189], indicating a likelihood of the removal of autoreactive long-lived B cells [186]. In another clinical study [190], all five patients receiving CD19 CAR-T therapy achieved remission for SLE according to the DORIS criteria.

In contrast to CAR-T, TCE can also be utilized in a short-term manner with the option of stopping treatment as needed. Compared to traditional IgG antibodies, TCE has the advantage of being able to leverage the T cells in germinal center and tertiary lymphoid tissue, and this advantage could be further reinforced by fbAbs with even lower molecular weight designs [191]. Clinical results [32,192] for compassionate use of blinatumomab for rheumatoid arthritis (RA) have shown decreased disease activity and reduced autoantibody levels. Improved synovitis was confirmed by both ultrasound and FAPI-PET-C (Fibroblast Activation Protein Inhibitor Positron Emission Tomography/Computed Tomography). Flow cytometry showed a reset with depletion of activated memory B cells, which were replaced by none-class-switched IgD-positive naïve B cells. Treatment was safe, characterized by a brief increase in body temperature and no signs of CRS [32,192].

Success has also been seen in a case report describing treating a patient with severe systemic sclerosis in a case report [193]. In the case report, a patient with severe systemic sclerosis experienced significant improvement in symptoms, characterized by a regained ability to move freely and resolution of burning skin sensations. By the end of the treatment, the patient had significant improvement in their condition. Interestingly, the treatment did not lead to an increased risk of infections. Serum IgG levels and specific antibody titers against infectious agents (haemophilus influenzae, pneumococcus) or toxins (tetanus and diphtheria) were not affected by the therapy [193], resembling the clinical observation of anti-CD19 CAR-T in treating SLE [190].

In alignment with targeting CD19, CLN-978, a CD3 × CD19 × serum albumin fbAb, is being investigated for a clinical trial in hematological malignancy [194] and SLE (NCT06613360), which has shown promising results as announced by the company and is being investigated for phase 1b trial for SLE [195]. Results in oncology [196] have shown that CLN-978 can effectively deplete B cells in B-non-Hodgkin Lymphoma, with a peripheral B cell depletion rate ranging from 93 to 98% within 96 h after a 30 μg subcutaneous dosing.

## 5. fbAb NK Cell Engagers (NKCE)

Natural killer cell engagers are specialized antibodies connecting with activating receptors, such as CD16a NKG2D, NKp30, NKp46 and NKG2C, as well as inhibitory receptors such as Siglec-7 [197], and are capable of inducing antibody-dependent cellular cytotoxicity (ADCC). Conventional antibodies, which activate the interaction of NK cells, exhibit weaker and more transient interaction with CD16a through its Fc domain [197,198].

Receptors on natural killer cells play crucial roles in the immune responses against tumors and viral infections. For instance, CD16a mediates binding to Fc of IgG format antibodies and significantly contributes to the efficacy of FDA-approved oncology antibodies, including rituximab, trastuzumab, and cetuximab. NKG2D (Natural Killer Group 2 member D) pairs with CD94 to respond to cytomegalovirus (CMV) infection by counteracting the inhibitory signal of NKG2A. NKp46 binds to CD3ζ or FcRγ (Fc receptor γ-chain) to mediate the lysis of target cells. NKp30 (Natural Killer protein 30) induces NK activation via interacting with B7-H6 [197,198]. Similar to T cells, NK can form an immunological synapse with the target cell via the bridging of antibodies, subsequently releasing cytotoxic molecules, such as granzyme B, perforin, IFN and TNF [197,198]. NK cells have two subsets: CD16^+^CD56^dim^ and CD16-CD56^bright^ subsets, where the former is primarily cytotoxic and the latter is associated with helper functions similar to CD4^+^ helper cells, and sometimes acts as regulatory NK cells by producing IL-10 [199].

Unlike non-engineered CD8^+^ T cells, which require recognition of peptides displayed on MHC-I molecules to perform cytotoxic functions, NK cells would kill MHC-I-negative cells, who are considered to be “non-self”. This feature serves as a complementary immune surveillance mechanism to T cells, allowing the detection of cancer cells or viral-infected cells that downregulate or inhibit the expression of MHC-I molecules to evade MHC-TCR-based T cell detection [200]. While NKCE holds promise, caution should be made, as there many debates regarding the therapeutic mechanism of NK cell engagers. When designing its NK cell engaging domain. For instance, the most popular target on NK, CD16A, is known to be cleaved by metalloproteases, such as ADAM17, after its activation, or by matrix metalloproteases, such as MMP25 [201]. Although some viewpoints are concerned that CD16A cleavage from NK cells may reduce the efficacy of NKCE, evidence from the clinically successful NKCE, AFM13 (CD16A × CD30), has shown that CD16A shedding could reduce the conjugation time between NK cells and target cells, which could in return increase the serial killing capacity of NK cells towards target tumor cells at distant site [202]. Moreover, CD16A has two polymorphisms, namely, the high-affinity variant 157V and 157F. Although the 157V CD16A variant NK cells generally exhibit higher preclinical efficacy in preclinical studies of CD16A-based NK cell engagers, roughly only 10% of the human population has homologs V/V variant of CD16A genes [203,204,205]. Therefore, for clinical relevance, polymorphism of CD16A should be considered for analytical relevance.

### 5.1. fbAb NKCE in Treating Cancer

NKCEs have gained popularity in oncology in both fbAb format (AFM28, AFM24, AFM13, GTB-3550, SAR443579/IPH6101) and IgG-like format (AFM26/RO7297089, AFVT-2101, TriNKET). While T cell engager clearly demonstrated efficacy in numerous trials, NKCE also possesses similar potential, if not superior efficacy and safety in certain cases. AFM28 (TandAb-Fc, CD16A × CD123) has achieved a composite complete response rate of 30% (n = 12) at dose levels of 250 mg and 300 mg against relapsed/refractory acute malignant leukemia (R/R AML) in a phase 1 trial [40,198]. Grade ≥ 3 treatment-related adverse effects (TRAE) were observed in 13% of patients. In comparison, phase I results of CD3 × CD123 T cell engager vibecotamab (XmAb14045) [206] showed an ORR of 11.5% in efficacy-evaluable group with 0.75 μg/kg or higher dose group (n = 87), with 87.5% (n = 120) of grade ≥ 3 TRAEs. In addition, combined NKCE and TCE therapies also hold promise in reducing cytokine secretion by T cells, as shown in a preclinical study targeting BCMA [207].

Moreover, NKCE can also be combined with immune checkpoint blockers for synergistic effects. AFM24 (TandAb-Fc, CD16A × EGFR), when combined with atezolizumab (480 mg AFM24 + 840 mg ate via infusion), achieved an ORR of 27% (4/15; 1 CR + 3 PR) in patients with WT-EGFR^+^ metastatic NSCLC with at least one prior therapy [208]. This efficacy is significantly higher compared to AFM24 monotherapy (1/10 of PR) [209] or atezolizumab monotherapy (ORR = 15%, [210]), suggesting a synergistic potential. Similar success was observed with AFM13 (TandAb, CD16A × CD30) when combined with pembrolizumab (ORR = 64.2–73.9%, [211]), achieving an ORR of 88% (n = 30) in relapsed/refractory Hodgkin lymphoma. However, the incidence rate of infusion-related reactions increased, necessitating the use of acetaminophen and corticosteroids [43]. AFM13, has also demonstrated monotherapy efficacy in relapsed/refractory peripheral T cell lymphoma (ORR = 32.4%, serious TRAE (treatment-related adverse events) = 8%, n = 108 [212]), and in relapse/refractory classical Hodgkin lymphoma (R/R HL) (ORR = 16.7%, n = 25, [213]). When combined with pembrolizumab, AFM13 achieves an ORR of 83% in relapsed/refractory Hodgkin lymphoma [43]. Given that cancer patients may undergo intensive pretreatments that compromise or exhaust conditions of NK cells, adoptive NK cell therapy is being clinically explored in combination with NKCE. For instance, AFM13 has been combined with allogenic NK cells (ab-101) in treating R/R HL [214], and AFM24 (CD16 × EGFR) with autologous SNK01 NK cells, which do not require lymphodepletion [215].

TriKE is a class of trispecific fbab, with a first domain targeting the NK cell antigen CD16, second cancer or viral antigen, and a third domain composed of an inserted IL-15 fragment to stimulate NK cells [74,75,76]. The CD16 × IL-15 × CD33 TriK, GTB-3550, was evaluated in a phase I trial and was shown to be able to control the disease progression (ORR = 0, disease control rate = 2/3, n = 3), with no clinically significant toxicity observed [77,78]. Researchers observed that the number of activated NK cells (CD69^+^) increased beginning on day 3 and remained above baseline through day 15 and day 22, until treatment completion [77,78]. To enhance therapeutic outcomes, GT Biopharama is developing a second-generation version of the drug, GTB-3650, which replaces the scFv domain of the drug with a camelid VHH. GTB-3650 has received FDA IND clearance, with a clinical study set to begin on 1 January 2025 [216].

Co-targeting CD16A and NKp46 is another approach to further enhance NK cell toxicity, as exemplified by the TriNKET as well as the ANKET platforms. TriNKET is a multispecific NK cell engager, targeting CD16a, NKp46, tumor-associated antigens, and in certain cases, the beta chain of the IL-2 receptor [62]. Unlike multivalent CD38-CD38 crosslinking by daratumumab, which may cause fratricide [217], preclinical studies have shown that CD16A-CD16A [217], or CD16-NKp46 crosslinking [61], does not cause fratricide. The safety profile of TriNKET DF1001 (CD16, NKp46, HER2) was favorable (incidence of grade 3 TRAE = 14%), and its efficacy (ORR = 13.9%, n = 36) suggests potential use in combination therapy for relapsed/refractory solid tumors [63]. Similarly, ANKET format antibodies developed by Innate Pharma and Sanofi have demonstrated promising preliminary clinical results. According to the company’s updated clinical result, the trispecific ANKET SAR443579/IPH6101 (CD16 × NKp46 × CD123), induced a complete response in 5 patients at 1 mg/kg dose in a phase 1/2 hematological malignancy trial [64].

### 5.2. fbAb NKCE in Treating Infectious Disease (Preclinical)

While no clinical-phase research antibodies are currently available, preclinical studies of NKCE have shown promise. NK cells are natural defenders against viral infection, capable of detecting MHC-downregulated target cells and engaging them via neutralizing antibodies via ADCC [218]. The BiKE CD16a × gp160/gp140/gp120 antibody [219] is constructed by fusing a single-domain soluble CD4 to an scFv. This antibody can bind to either coated gp140sc (gp140, soluble and cleaved), gp160sc-expressing 293 T cells, or gp160-expressing CHO cells. gp160 is cleaved into gp120 and gp41, whereas gp140 is an engineered version of gp160 that lacks gp41 so it is more soluble and easier to study for experimental purposes [220]. These antibodies can induce NK cell degranulation and killing of Env-expressing cells, as well as the elimination of both chronically and acutely HIV-infected T cells by PBMC. Due to their small size, these antibodies may also be capable of crossing the blood-brain barrier (BBB) and achieving deep tissue removal of HIV-infected cells.

### 5.3. fbAb NKCE in Treating Autoimmune Disease

The current literature suggests a limited number of clinical phases NKCE developed for the treatment of autoimmune disease is available. Nevertheless, insights from allogenic and engineered NK (CAR-NK) can provide valuable clinical prospects. Analogous to the concept of CD19 CAR-T, the allogenic CD19 CAR-NK, named NKX019, when combined with rituximab or obinutuzumab, can synergistically deplete pathogenic B cells [221]. As a result, NKX019 is currently being evaluated in a phase I trial for systemic lupus erythematosus (SLE) requiring lymphodepletion [222]. As treatment with immune cell engagers does not require lymphodepletion, it is possible that NKCE poses a competitive edge over CAR-NK for treating autoimmune disease.

This observation suggests not only the feasibility of combining CD19 and CD20 as combination targets for treating lupus but also that immune cell engagers may potentially be combined with CAR-NK for enhanced therapeutic outcomes. Preclinical research has shown that combining CD19-CAR-NK and rituximab (anti-CD20) can help to overcome CD19-antigen, preferentially sparing CD19^+^ healthy B cells (versus cancer cells), and boost CAR-NK degranulation and cytokine production capacity [223]. In addition, an in vitro study has shown that combining CD19 antibody with CD19 CAR-NK or CAR-T can enhance the serial killing capacity of these immune cells by facilitating rapid detachment of these CAR effector cells from target cells by reducing trogocytosis [224], further testifying the validity of combined B-cell depleting immunotherapy in treating autoimmune disease. Similarly, iPSC (Induced Pluripotent Stem)-derived CD19-CAR-NK, such as FT522, is armed with features such as anti-CD19 CAR, non-cleavable CD16 (binding to therapeutic antibody), and IL-15/IL-15 receptor, CD38 knockout to increase mitochondrial respiratory capacity. FT522 has demonstrated B cell-depleting capacity in oncology when combined with rituximab [225].

## 6. Myeloid Cell Engagers (MCE) and B Cell Engager (BCE)

Besides T cells and NK cells, targeting myeloid cells can not only address the specific therapeutic needs but also potentially create synergistic effects with other immunotherapies. Due to the overlapping of some cell surface receptors between myeloid and B cells, such as CD40 and TLRs, myeloid cell engagers can sometimes activate both cell types, leading to additive efficacy. As of 2024, there are eight MCEs in clinical trials, although five MCEs have been discontinued from further clinical development, highlighting the opportunities and challenges in this field [82,198]. Numerous macrophage and neutrophil engagers (FcγRI (Fc-gamma receptor I), FcαRI (Fc fragment of IgA receptor), SIRPα (Signal Regulatory Protein alpha)), dendritic cell engagers (CD40), granulocyte engagers, and B-cell engagers, which are identified, studied and put for clinical trial [82,198].

Macrophages, derived from circulating monocytes, perform phagocytosis, engulfing targets such as cell debris, cancer cells, or pathogens. Phagocytosis can be facilitated through antibody bridging, a process known as antibody-dependent cellular phagocytosis (ADCP) [82,198]. Neutrophils are usually recruited to sites of infection or tumorigenesis, where they perform degranulation, phagocytosis, and trogocytosis. Both macrophages and neutrophils can exhibit proinflammatory/antitumor phenotype or immunosuppressive/protumorigenic phenotype [82,198]. B cells, in contrast, produce antibodies and coordinate with T cells, which can play therapeutic roles in targeting pathogens and tumors or pathogenic roles in generating autoreactive immunity.

The discontinuation of the development of many myeloid cell engagers (MCEs) was not due to the drugs themselves. Many clinical trials for these MCEs were performed in the early 2000s when limitations in clinical understanding and biomedical technologies hindered the development of these agents. At that time, the lack of modern cytokine release syndrome (CRS) management protocols and anti-IL-6 drugs like tocilizumab (approved in 2017 [226]) prevented the developers from effectively mitigating safety concerns. In addition, early clinical trials often lack biomarker-driven patient selection. For instance, the phase II trial of MDX-H210 [227] included patients with varying HER2 expression levels, and the trial for MDX-447 [228] also did not require EGFR positivity from in patient. Moreover, the immature state of recombinant technologies and the high cost of synthetic biological production at the time presented challenges for manufacturing multispecific antibodies. The rise of immune checkpoint inhibitor drugs has diverted corporate resources away from MCEs, leading to pipeline discontinuation. Macrophages, which express all classes of FcγR receptors and FcαR receptors can execute ADCP, ADCC, and mediation of inflammation and cytokine release [82,198]. With these earlier roadblocks now largely overcome, MCE R&D may experience a resurgence.

Many clinical phase MCEs were examined in clinical trials [82,198], including but not limited to: MDX-210/MDX-H210 (FcγRI/CD64 × HER-2 [227,229,230]), MDX-447 (FcγRI × EGFR [228]), H22 × Ki4 (FcγRI × CD30 [231]), BDC-1001 (TLR7/8 × HER2 [232,233]), NJH395 (TLR7 × HER2 [234,235]), TAC-001 (TLR9 × CD22 [236,237]), BR105 (SIRPα with silenced Fc [238]), IMB071703 (4-1BB/CD40L [239]), MGD010 (DART-Fc, CD32B × CD79B [240]), and MP0317 (the only clinical phase DARPin fbAb, CD40 × FAP [241,242]). These antibodies incorporate scFvs to bind to the antigens or employ covalent fusion to protein ligands or small-molecule agonists, collective referred to as immune-stimulating antibody conjugates (ISACs), resulting in a diverse spectrum of myeloid cell engagers.

### 6.1. MCE and BCE in Treating Cancer

FcγRI/CD64-based, antibodies, such as MDX-210/MDX-H210, MDX-447, and H22 × Ki4, can activate macrophage, monocytes, dendritic cells, and cytokine-primed neutrophils by binding to IgG, thereby initiating ADCC and ADCP [82,198]. Since these drugs were tested during clinical trials in the early 2000s, their limitations were highlighted by difficulties in managing drug-related symptoms. Overall, moderate or no efficacies and only transient responses were reported in these trials [227,228,229,230,231]. One of the challenges in targeting FcγRI is that serum IgG can compete for CD64 binding, thereby reducing efficacy through competitive inhibition [243]. Engineering of these antibodies to target different epitopes may resolve this issue. Alternatively, co-activating both FcγRI and FcαRI [244] could further enhance macrophage potency and coordinate cytotoxicity with NK cells.

CD40-targeted therapies, including IMB071703 (4-1BB/CD40L [239]) and the DARPin format fbAb MP0317 (CD40 × FAP [241,242]), function by orchestrating interactions between T cells and antigen-presenting cells (APCs). IMB071703 targets 4-1BB on T cells and CD40 on APCs (including B cells), particularly dendritic cells, promoting immune synapse formation and facilitating crosstalk between the cells to activate antigen-specific adaptive immune response. However, clinical trials showed no objective response [239], in contrast to promising outcomes observed in preclinical studies [60]. The dosing regimen, which involved intratumoral injection, may not present an optimal pharmacokinetic profile as indicated by its clinical half-life of 2.45 h [241,242]. Infusion or subcutaneous route (as discussed in the previous sections for T cell engagers), may offer more sustained stimulation and a higher cumulative chance of eliciting adaptive immunity.

MP0317, on the other hand, activates CD40^+^ APCs via its anti-FAP arm, which recognizes fibroblasts in the tumor microenvironment, therefore limiting toxicity to fibroblast-rich tumors. Phase I studies [241,242] demonstrated good tolerability in all dose groups (0.01–10 mg/kg). Tumor biopsies confirmed successful co-localization of MP0317 with FAP and CD40. Genes associated with B cell trafficking (CXCL3, CXCR5, CCR6, and CCL20) and IFN-γ downstream chemokines (CXCL9, CXCL10) were upregulated, while pro-inflammatory genes (IL-2, IL-6, IL-8) were not, indicating tumor microenvironment remodeling [241,242].

TLR7/8 and TLR/9 are expressed in myeloid cells and B cells [82]. These receptors play key roles in pathogen recognition, particularly of ssRNA (single-stranded RNA) and CpG DNA. Due to their small molecular size, TLR ligands can be covalently linked to fbAb to create anti-TLR functionality. TLR7 promotes the production of type I interferons (IFN-α/β) and dendritic cell maturation [245], while TLR8 can activate activates NF-κB and induces proinflammatory cytokines (e.g., TNF-α, IL-12, IL-1β) that recruits neutrophils and macrophages [246,247]. TLR9 triggers interferon regulatory factors and NF-κB pathways, regulating B cell tolerance and potentially suppressing TLR7-driven autoantibodies in lupus models [246,248].

Recent clinical development of TLR-targeting antibodies differs from early MCE trials by taking advantage of combinatory therapy. For example, the phase I/II trial of BDC-1001 (TLR7/8 × HER2) included co-treatment with nivolumab [232], demonstrating good tolerability and efficacy in heavily pretreated patients. NJH395 (TLR7 × HER2), a first-in-class ISAC [234] showed a manageable safety profile in clinical trials, although neuroinflammation was observed at high doses, suggesting potential interaction with CNS-resident myeloid cells. Anti-drug antibodies were detected in all tested patients, potentially limiting repeat dosing. Non-linear pharmacokinetics indicated target-mediated drug disposition. While both BDC-1001 and NJH395 targets HER2 and TLR, NJH395 specifically targets TLR7, thereby inducing IFN-α/β without IL-1β induction via TLR8, which may improve efficacy at the cost of increased systemic toxicity [249]. Due to insufficient efficacy, both BDC-1001 [250] and NJH395 [251] were terminated for further development.

TAC-001 is a TLR9 × CD22 antibody that significantly activates both B and T cells, including immunoglobin secretion, and is currently under investigation in a phase I/II trial [236,237]. In addition to its oncology application, TAC-001 has also shown promise in enhancing vaccination efficacy in preclinical studies involving elderly animal models [252]. For reference, this review [249] provides a comprehensive summary of TLR-based drug clinical trials in cancer therapy.

Both macrophages and neutrophils express the SIRPα receptor, which binds to CD47, a receptor that can inhibit phagocytosis (known as the “don’t eat me signal”) [249]. Thus, blocking SIRPα-CD47 interactions may promote phagocytosis, leading to both tumor cell clearance and enhanced antigen presentation. BR105 is an anti-SIRPα antibody with a silenced Fc region [238]. Phase I clinical results show no dose-limiting toxicities, although all patients experienced treatment-related adverse events. However, no treatment-related discontinuation or deaths occurred. Pre-clinical studies [253] showed that BR105 binds to various SIRPα variants, synergizes with therapeutic antibodies to promote phagocytosis, and inhibits tumor growth in vivo.

Activation of STING (stimulator of interferon genes) can trigger interferon expression, enhancing the efficacy of cancer therapies including immune checkpoint and radiation therapy, by sensitizing myeloid cells [254]. TAK-500 [254], a STING agonist, activates CCR2^+^ myeloid cells in the tumor microenvironment and converts immune suppressive “cold tumor” into “hot tumor” characterized by CD8^+^ T cell proliferation and activation [254,255]. TAK-500 is currently being evaluated in combination with pembrolizumab in clinical trials [255].

### 6.2. MCE and BCE in Treating Infectious Disease

MDX-240 (FcγRI × gp41) is a clinical-stage antibody aimed to target HIV gp41, and its clinical development was terminated, with very limited data available for reference [82]. While the reasons behind the termination of clinical trials might be similar to those of other myeloid cell engagers tested from the 1990s to 2000s for cancer treatment (see the previous section), preclinical data indicate that MDX-240 could reduce (but not eliminate) reverse transcriptase activity in cell culture, even in macrophage with active infection, demonstrating efficacy. However, the antibody alone is not likely to be sufficient for treating HIV. Cocktail approaches, involving cytotoxicity and coordination of multiple immune cells are most likely required. Research [256] has shown that antibodies from AIDS patients who are HIV controllers and untreated progressors exhibited enhanced phagocytic activity compared to treated patients. In these patients, antibodies demonstrated a potential to bind to FcγR2a (CD32a), an activating receptor. Blocking the inhibitory FcγR2a receptor has no effect on the phagocytosis of HIV antibodies from chronically infected patients but increased phagocytosis from antibodies derived from controllers, indicates that antibodies from patients at different pathological stages can target different Fc gamma receptors, and that specifically targeting activating receptors of myeloid cells can potentially improve treatment efficacy.

While engineering high-affinity FcγR-targeting antibodies may be considered as advantageous for combating viral infection, a phenomenon known as antibody-dependent enhancement (ADE) is frequently hijacked by viruses to facilitate entry into and infection of immune cells through crosslinking of the virus and the immune cell by the antibody. Dengue virus has been shown to exhibit greater severity at lower serum antibody concentrations, and reducing antibody binding affinity to FcγRs may help to mitigate this issue [257]. Myeloid cells expressing FcγR, especially macrophages and dendritic cells, are particularly susceptible to this process [258,259]. Pathogens like dengue virus, Zika virus, coronavirus, HIV, and RSV are known to induce ADE, although the mechanism of action differs [259]. For instance, ADE caused by dengue virus arises due to the inability of the antibody to neutralize heterologous serotype, resulting in binding without neutralization. Coronavirus-induced ADE is attributed to conformational changes in the spike protein, facilitating viral entry via canonical receptor-dependent pathways involving the receptor binding domain. HIV can exploit the antibodies to the surface of target cells, promoting membrane fusion via FcRs and complement receptors. In some cases, IgA from HIV-infected patients can mediate and promote IV infection of primary human monocytes [259,260,261].

### 6.3. MCE and BCE in Treating Autoimmune Disease

Crosstalk between APC cells B cells and T cells plays a key role in inducing autoimmunity. APCs can present endogenous antigens to helper T cells, leading to the activation and proliferation of autoreactive T cell clones. Blocking the interaction between CD28 and CD80/CD86 may reduce T-cell activation. One of the most studied antibodies is abatacept, an FDA-approved anti-CD80/CD86 antibody, which functions by inhibiting the binding between T cells and APCs. It has been investigated for the treatment of RA, juvenile idiopathic arthritis, psoriatic arthritis, and prophylaxis of acute graft-versus-host diseases. Clinical data suggest that abatacept can not only treat patients with rheumatoid arthritis [262], but also help reduce the progression of high-risk patients into rheumatoid arthritis in high-risk individuals [263]. In addition, although clinical trials with shorter treatment duration for Sjögren’s syndrome (SS) have shown limited efficacy [264], longer treatment duration demonstrated significant therapeutic benefit [265].

In addition to targeting myeloid cells, targeting CD40-CD40L interaction between T cells, B cells, and APC cells has proven efficacious in treating autoimmune diseases. CD40L is expressed on activated T cells and platelets, whereas CD40 is expressed on myeloid cells and B cells. Disrupting this crosstalk reduces the ability of B cells to mount antigenic responses to endogenous antigens, and it hinders T cell-APC interactions, holding promise for reducing the generation of adaptive autoimmunity [266].

The CD40L agonist dapirolizumab pegol (DZP), a pegylated Fab antibody, is being developed to treat SLE. A preclinical study [266] of DZP (known as CDP7657) demonstrated that DZP could inhibit antigen-specific immune responses to tetanus toxoid in cynomolgus monkeys, indicating DZP’s ability to reduce antigenic response that contributes to autoimmunity. Importantly, the antibody did not induce thrombotic complications, a key safety consideration, as CD40L is also expressed on platelets [266]. DZP has a high affinity of 7.9 pM for CD40L, enabling engaging with its ligand [267]. Although DZP shows efficacy, its phase 2 study [267] revealed that withdrawal of the drug can lead to worsening of disease symptoms. In its phase 3 results [268], DZP combined with the standard of care (SOC) led to a 60.1% (n = 208) response rate at at week 48 according to SRI-4, compared to 41.1% in the placebo + SOC group (n = 107; *p* = 0.0014). (Severe BILAG flares occurred in 11.6% of patients receiving DZP + SOC, versus 23.4% in the placebo + SOC group by week 48 (*p* = 0.0257)). Patients also experienced reductions in corticosteroid doses following DZP treatment, and serious adverse events were less frequent in the DZP group compared to placebo (9.9% vs. 14.8%).

As mentioned in Section 6.1, activation of TLRs (toll-like receptors) have been exploited for their therapeutic functions in treating cancer. In autoimmune diseases, activation of TLRs could be unwanted, as it promotes the pathogenic process of autoimmune conditions. For instance, TLR4 plays a role in rheumatoid arthritis (RA) and antiphospholipid syndrome (APS). In APS, TLR4 signaling is needed for the activation of B cells producing autoreactive antibodies [269]. In RA, anti-citrullinated protein antibodies (ACPAs) are characteristic of RA and may serve as a prognostic marker of the disease. Specifically, ACPAs and citrullinated proteins can form a complex that can activate TLRs, especially TLR4 on immune cells and stromal cells [270]. Nevertheless, the result of the clinical trial of an anti-TLR4 inhibitory antibody, NI-0101 [270], has shown that blocking TLR4 alone cannot significantly improve RA symptoms, for a patient who has established RA and has inadequate response from methotrexate. Broader spectrum approaches targeting the TLR family may be needed to achieve substantial efficacy.

Inhibiting B cells is another strategy to modulate autoimmune disease. By blocking B cell signaling activation, it is possible to inhibit downstream pathways responsible for autoantibody production. MGD010 is a DART-Fc antibody that targets CD32B and CD79B. CD32B is an inhibitory receptor for B cells, and CD79B is a component of the B cell receptor complex; crosslinking these two receptors can suppress B cell activity without depleting the cells. Clinical evidence suggests that MGD010 could reduce B cell activation, as indicated by decreased BCR-induced Ca^2+^ mobilization and sustained downregulation of surface BCRs on circulating CD27^+^ memory B cells [240]. The safety profile of MGD010 has also been confirmed, with all adverse events being below grade 3 and no severe adverse effects reported [240].

## 7. Immune Checkpoint Blocker (ICB) and Immune Checkpoint Agonist (ICA)

As shown by FDA approvals, immune checkpoint blockers target PD-1 (Nivolumab, pembrolizumab, cemiplimab), PD-L1 (atezolizumab, avelumab, durvalumab), and CTLA-4 (ipilimumab). CTLA-4 can negatively regulate T-cell activation via competitive binding to the ligands of CD28, which are CD80 and CD86. Anti-CTLA-4, by preserving CD28 binding free of CTLA-4 interference, serves as a preserver for CD28 signaling in T cells [80]. Moreover, if equipped with Fc, such as ipilimumab, such antibodies can mediate ADCC to lyse CTLA-4^+^ regulatory T cells [271], which can be beneficial for treating cancer and infectious diseases but potentially harmful for autoimmune conditions. PD-L1 is expressed on macrophages, dendritic cells, B cells, and T cells, whereas PD-L1 and PD-L2 are expressed on both antigen-presenting cells and cancer cells [80]. Activation of the PD-1/PD-L1 axis can blunt T-cell inflammatory responses and restrict their proliferation after activation [80]. While both anti-PD-1 and anti-PD-L1 antibodies can block the PD-1/PD-L1 interactions, anti-PD-L1 possesses the additional feature of freeing CD80 from PD-L1 binding, thereby freeing additional CD80^+^ dendritic cells [80]. In terms of biological differences, CTLA-4 is expressed early during T cell activation and primes T cells in lymphoid tissue, whereas PD-1 is largely expressed at later stages of T cell activation and functions predominantly in peripheral tissues to limit T cell activity in inflammation or infection. Besides targeting PD(L)1 and CTLA-4 (Cytotoxic T lymphocyte antigen 4), ICBs that target LAG-3 (Lymphocyte Activation Gene 3), TIGIT (T-cell immunoreceptor with Ig and ITIM domains), and TIM-3 (T cell immunoglobulin and mucin domain-containing molecule-3) are also under clinical development [272]. Fragment-based immune cell engagers can also circumvent the need for Fc silencing, which can otherwise lead to phagocytosis of T cells by macrophages via Fc-PD-1 mediated binding [273].

Similar to treating cancer, the aim of ICB in treating infectious diseases has been strategically focused on reinvigorating or preventing the occurrence of exhausted immune cells, as well as counteracting evasion or inhibition of immunity by pathogens [274,275]. Chronic infections, such as HIV, HBV, HCV malaria, and tuberculosis (TB) can lead to upregulation of immune checkpoint marker expression, as they can induce T cell exhaustion [276].

As CD4^+^ T cells with exhaustion markers (PD-1, LAG-3, and TIGIT) have been shown to contain HIV DNA at a higher frequency, these targets of ICBs are also biomarkers, serving as good targets for eliminating viral reservoirs [272]. In HIV treatment, the effect of immune checkpoint blockers displayed mixed outcomes and should be considered with caution. Both reversal of viral latency/reactivation of virus due to pembrolizumab (PD-1) treatment [277], and suppression of viral rebound from budigalimab (PD-1) treatment [278], have been reported. Such conflicting results highlight a need to investigate the mechanisms of ICB in virology research.

In the case of budigalimab, delayed rebound of HIV RNA was observed [278]. Oncological clinical studies of budigalimab [279] have shown that budigalimab exhibits similar efficacy (ORR = 13% for HNSCC (Head and Neck Squamous Cell Carcinoma) and 19% for the NSCLC (Non-Small Cell Lung Cancer)) compared with pembrolizumab (ORR = 18% for NSCLC). Budigalimab has the L234A/L235A (LALA) IgG1 mutation in the Fc domain, whereas pembrolizumab contains an IgG4 S228P mutation in the Fc [280]. It has been shown that while the S228P mutation poses negligible impact on binding to Fc gamma receptors, the L234A/L235A mutation can significantly reduce binding to Fc gamma receptors [281], suggesting potential impact from Fc-dependent modalities. For pembrolizumab, a study [282] utilizing patient CD4^+^ T cells with latent HIV infection has shown that PD-1 signaling is able to suppress the T-cell receptor (TCR) induced P-TEFb (Positive Transcription Elongation Factor b) pathway, a key regulator for HIV transcription. Inhibition of PD-1 with pembrolizumab can remove such inhibitory signal, restoring the function of P-TEFb as well as promoting the transcription of HIV RNA. Increased unspliced HIV RNA and plasma viremia, but not HIV specific T cell responses to clear reactivated cells, were induced by pembrolizumab, even while ART was not interrupted [277].

Similarly, HBV, HCV, and EBV-infected patients have upregulated PD-1 levels, whereas in acute HBV PD-1 is upregulated, in chronic HBV conditions, PD-L1, TIM-3, and CTLA-4 are also upregulated [275]. In parasitic malaria infections, due to the recurrence of infection where multiple infections are needed for conferring immunity, continuous antigenic exposure can cause immunosuppression, through upregulation of PD-L1, LAG-3, CTLA-4, OX40, and TIM-3 [274,275]. Mice lacking PD-1 can clear infection of a usually chronic strain of malaria [283]. In bacterial infection from M. *tuberculosis*, H. *pylori*, and L. *donovani*, PD-1 levels are also increased [274,275]. However, mouse studies also suggest that mice lacking PD-1 can suffer from dramatically higher mortality rates from TB, while other cases suggest that PD-1 can reduce interferon production to avoid immune-mediated tissue damage, suggesting the protective roles of PD-1 in some diseases [275].

Clinical observations of oncological treatments with ICBs have led to interest in applying ICBs in treating autoimmune diseases. The incidence of immune-related adverse events (irAEs) is a prominent side effect of ICBs, caused by overriding self-tolerance signals, such as PD-1, that are expressed on healthy tissues and organs [284]. While ICBs used in oncology primarily serve to remove inhibitory signals, in autoimmune diseases, immune checkpoint agonists (ICAs) could be used to suppress pathogenic autoimmunity [285]. Various PD-1 and CTLA-4-targeting antibodies are being evaluated and placed for clinical trials. Phase I trials like anti-PD-1 rosnilimab [286] have been shown to induce a higher Treg to Teff (effector T cell) ratio as well as a reduction in interferon-gamma levels. According to the company’s announcement [287], rosnilimab has achieved significant responses by week 14 for moderate to severe rheumatoid arthritis based on ACR20 (American College of Rheumatology 20 standard or 20% improvement from baseline of disease) and ACR50 standard. Phase II trial of anti-PD-1 peresolimab [288] has shown improvement in patients with rheumatoid arthritis (RA) according to the ACR20 standard at 700 mg dose group.

To maximize the efficacy of blocking immune checkpoints, co-targeting multiple immune checkpoints is also a very promising strategy. Specifically, a multi-targeting approach could more selectively target immune cell populations that express two or more of the desired checkpoint targets. Interestingly, multispecific immune checkpoint antibodies have the potential to induce fewer adverse effects compared to combinations of multiple monospecific immune checkpoint blockers. For instance, according to a metanalysis [289,290], combined monotherapies regimens of CTLA-4 (lpilimumab) and PD-1/PD (pembrolizumab or nivolumab) can cause incidence rate of irAEs for 35–47% (≥grade 3: 13–15%) with sequential dosing and irAEs of 60–83% (≥grade 3: 27.5–36%) for simultaneous dosing. In comparison, for bispecific PD-1/CTLA-4 antibody AK104 in a phase II trial [291], the rate of irAE is 53.3% (≥grade 3: 17.8%), which is comparable to the irAE of sequential dosing and less toxic than that of simultaneous dosing.

Similarly, the clinical progress for other co-stimulatory antibodies, such as the CTLA-4 × PD-1 AK104, MEDI5752, XmAb20717, as well as the PD-1 × LAG-3 tebotelimab (DART-Fc, also known as MGD013), PD-L1 × CTLA-4 lorigerlimab (DART-Fc, also known as MGD019) and PD-1 × TIM-3 RO7121661 [38,289], has been encouraging. In its phase I trial against both solid tumor and hematological malignancies [36], tebotelimab induced tumor shrinkage in 34% of patients, including patients with PD-1 refractory disease, LAG3^+^ non-Hodgkin’s lymphoma, and CAR-T refractory disease. Clinical responses were also observed in patients who were unresponsive to anti-HER2 and anti-PD-1 combination therapy. Lorigerlimab achieved an ORR of 25.7%, with 32.3% of irAEs and 7.9% of ≥grade 3 irAE in the treatment of metastatic castration-resistant prostate cancer (mCRPC) [38].

The VHH-Fc-based antibody PD-L1 × CTLA-4 erfonrilimab (KN046) has achieved promising results for clinical trial, as monotherapy or combination therapy with chemotherapy or targeted therapy [292,293,294,295,296], It has been tested as first-line therapy for metastatic triple-negative breast cancer as first-line therapy in combination with nab-paclitaxel (ORR = 44%) [293], hepatocellular carcinoma in combination with lenvatinib (ORR = 51.9%) [295], pancreatic cancer as combination with nab-paclitaxel and gemcitabine [297] (ORR = 45.2%), and nasopharyngeal carcinoma (ORR = 12.5%) [292]. Interestingly, the design of erfonrilimab preserves the Fc function, allowing Fc gamma receptor-mediated effector functions such as ADCC and CDC, which in turn regulate T cells in the tumor microenvironment [292].

Importantly, erfonrilimab serves as an example demonstrating that a multispecificity fashion of designing an immune checkpoint blocker is capable of exerting higher efficacy than combination therapies. In the treatment of metastatic non-small cell lung cancer, erfonrilimab with pemetrexed as first-line therapy achieved an ORR = 46% and a median OS of 26.6 months [294]. In comparison to other cocktails of Anti-PD-L1 with CTLA-4 trial, combining nivolumab plus ipilimumab yielded an ORR of 33.4% with median OS of 17.1 months in the CheckMate 227 trial and an ORR of 38.2% and median OS of 15.6 months in the CheckMate 9LA trial, while co-treating durvalumab, tremelimumab, and chemotherapy yielded an ORR of 38.8% and median OS of 14 months in the POSEIDON trial [294].

## 8. Conclusions

In the new era of immunotherapy, pharmacogenomics and precision medicine can assist physicians in making more effective and personalized treatment plans for patients. Therapies like CAR-T and neoantigen vaccines represent major therapeutic alternatives to immune cell engagers. While these emerging therapeutics are being proven to be life-saving black horses, they pose significant challenges in competition with antibody therapeutics, including all immune cell engagers.

Immune cell engagers have an advantage by themselves compared to CAR-T or vaccines/neo-antigen vaccines, including but not limited to: (1) no need to be tailored or individualized, (2) lower cost, (3) can be easily applied as combination therapy. While each immune cell engager presents uniqueness and promising aspects, a large portion of clinical results has indicated that using these immune cell engagers alone cannot achieve curative effects. Specifically, mono-therapeutic immune cell engagers are challenged by (1) the limited presence of effector immune cells at the site(s) of disease, (2) antigen escape by target cells or pathogens, and (3) cannot directly establish permanent adaptive immunity activation (cancer and infectious diseases) or inhibition (autoimmunity). These challenges could be potentially resolved by cocktail therapy composed of multiple classes of immune cell engager, so as to mobilize the immune system and achieve therapeutically meaningful crosstalks. Extended half-life can be a crucial factor in the oncology field, as patients need sustained exposure to the drug for efficacy. Shortening the half-life of a drug could be beneficial in treating autoimmune diseases and infectious diseases when the disease condition can be rapidly resolved by transient medication.

TCEs are the most clinically studied category of fragment-based immune cell engagers for now. Lessons from TCE could be applicable to other immune cell engagers as well. Plenty of efforts, including antibody engineering and dosing regimen/route optimization, have supported modulation of its plasma concentration to achieve optimized efficacy, toxicity, and reduced exhaustion of immune cells. Emerging efforts have shown that immune checkpoint blockers could be either co-treated or incorporated as a submodule of fbAb TCE. The application of TCE in HIV has shown some promising outcomes but its ability to target viral-dormant cells and target antigen escape still needs further evaluation. In the treatment of autoimmune disease, by eradicating B cells, TCE has shown clinical efficacy. A longer period of follow-up and comparison with CAR-T could facilitate the understanding and development of TCE.

Like TCE, Fragment-based NKCE holds potential. While the safety profile of fragment-based NKCE is widely acknowledged, its efficacy as monotherapy presents a challenge. The efficacy of NKCE could be further improved with the presence of immune checkpoint blockers as well as other approved drugs, highlighting the need for further translational research. The role of NKCE in autoimmune disease and infectious disease is under-explored, which contrasts with the higher popularity of CAR-NK therapies in the field. Myeloid cell engagers are regaining popularity in clinical development but may entail a more effective trial design approach to achieve substantial efficacy. Simultaneously targeting multiple myeloid cell receptors may be necessitated to achieve meaningful efficacy. Immune checkpoint blockers are frequently combined with other immune cell engagers. Combinations of multiple immune checkpoint blockers, either by co-administering two separate antibodies or by administering multivalent variants, hold promise to achieve higher efficacy. Cancer, infectious disease, and autoimmune disorders mark three of the primary areas of focus in immunotherapy. The crosstalk of pathways and strategies for treating these diseases motivates researchers to investigate and develop more efficacious drugs. Recent advancements in AI, machine learning, multi-omics, ex vivo organoid systems, and high throughput methods, have significantly accelerated the pace of drug discovery and development. While these tools can miraculously shorten the time required to obtain results, the difficulty of critical evaluation could increase as the volume of data researchers must process continues to grow. The translatability of drug research is complex, as highlighted by challenges such as anti-drug antibody formation, pharmacokinetics, efficacy, and unexpected toxicities (Table 1). These issues underscore the importance of examining such traits during the preclinical phase and comparing them with clinical-stage or approved drug products, to prudently assess their competitive edge from a business and humanitarian perspective.

The global economy has remained at a nadir since the onset of COVID-19. Reduced availability of grants and funding has compelled both industry and academia to adopt stricter criteria for approving or terminating drug R&D efforts. Thus, the ability to critically evaluate the clinical potential of drug candidates is more essential than ever. As more clinical trial results become available, learning from the past and anticipating issues in future clinical phases can serve as a guiding light for navigating the future of immune cell engagers in this era of uncertainty.

## Figures and Tables

**Table 1:** Summary of clinical results for fragment-based immune cell engagers discussed in this review. Abbreviations: ADA: anti-drug antibody, HLA: human leukocyte antigen, TEAE: treatment-emergent adverse events, TRAE: treatment-related adverse events, SAE: serious adverse events, AE: adverse events. CR: complete response rate, ORR: overall response, PK: pharmacokinetics, SC: subcutaneous (route), cIV: continuous IV infusion, PR: partial response, SD: stable disease, AML: acute myeloid leukemia, ALL: acute lymphoblastic leukemia, MDS: myelodysplastic syndromes, SCLC: small cell lung cancer, NSCLC: non-small cell lung cancer, NEC: neuroendocrine carcinoma, SRI-4: reduction in the SLE Disease Activity Index (SLEDAI) by ≥4 points. Note: Data for incidence of ADA and half-life may come from a different source of trial, rather than the source of trial for efficacy and adverse events (with patient size indicated).

Antibody Characteristic								Clinical Result				Reference
Antibody Name(s)	Format	Half-Life	Incidence of ADA	Disease	Target(s)	Development Stage	Prospect	Patient Sample Size (N)	Efficacy/Outcome	≥Grade 3 Events		Reference
T cell engager (Infectious disease)												
IMC-M113V	ImmTAV	N/A	N/A	AIDS/HIV	CD3 × gag (HLA-A*02:01)	phase 1/2 ongoing	ongoing development	16	Dose-dependent delayed viral rebound and/or viremia control (18.8%)	“no serious adverse events”		[298]
IMC-I109V	ImmTAV	N/A	N/A	Chronic HBV infection	CD3 × env (HBV; HLA-A*02:01)	phase 1 ongoing	ongoing development	21	decline of serum viral surface antigen level (HBsAg); elevation in ALT and IL-6	0%		[155]
MGD014	DART-Fc	12 d	8.30%	AIDS/HIV	CD3 × gp120	phase I completed	ongoing development	24	N/A	“well tolerated”		[177]
Myeloid cell engager and B cell engager (infectious disease)												
MDX-240	bispecific	N/A	N/A	AIDS/HIV	FcγRI × gp41	Clinical phase	development terminated	N/A	efficacy unknown; reduce infectivity in human monocyte-derived macrophages	N/A		[82]
T cell engager (autoimmune disease)												
Blinatumomab	BiTE	N/A	N/A	multidrug-resistant rheumatoid arthritis (RA)	CD3 × CD19	clinical (compassionate use)	ongoing development	6	Sustained remission at month3; reduced autoantibody level	“no serious adverse events”		[32,192]
Blinatumomab	BiTE	N/A	N/A	severe systemic sclerosis	CD3 × CD19	case report	possible development	1	“significant improvement of symptom, regained ability to move”	“well tolerated”		[193]
CLN-978	BiTE-albumin	N/A	N/A	systemic lupus erythematosus (SLE)	CD3 × CD19 × albumin	phase 1b ongoing	ongoing development	2	N/A	N/A		[196]
Myeloid cell engager and B cell engager (autoimmune disease)												
MGD010	DART-Fc	8 d	N/A	autoimmunity	CD32B × CD79B	phase I completed	Collaborative development terminated	8	“decreased B cell activation with no signs of B-cell depletion”	0%		[240]
dapirolizumab pegol	Fab (PEG)	N/A	N/A	autoimmunity	CD40L	phase 3 completed	ongoing development	208	60.1% SRI-4 vs. control (41.1%)	9.9% (TEAE)		[268,299]
antibody characteristic								clinical result				reference
antibody name(s)	format	half-life	incidence of ADA	disease	target(s)	development stage	prospect	patient size (N)	ORR	CR	≥grade 3 events	reference [1]
T cell engager (oncology)												
blinatumomab	BiTE	1.41–2.10 h	2%	hematological malignancy	CD3 × CD19	FDA-approved	FDA-approved	1373	N/A	54%	80%	[28,97]
blinatumomab + nivolumab or ipilimumab	BiTE	N/A	N/A	Relapsed/Refractory (R/R) ALL	CD3 × CD19; CTLA-4; PD-L1	phase 1 completed	ongoing clinical development	27	N/A	68%	N/A	[300]
duvortuxizumab/MGD011/JNJ-64052781	DART-Fc (mut)	7.50%	N/A	B cell malignancies	CD3 × CD19	phase 2 completed	discontinued; neurotoxicity and lack of commercial competitiveness	N/A	“some patients”	N/A	“similar to those of other CD19 TCEs”	[101,301,302,303]
AFM11	TandAb	18.4–22.9 h	N/A	B cell malignancies	CD3 × CD19	phase 1 completed	discontinued; neurotoxicity and limited efficacy	33	9.10%	0%	27.30%	[41,42]
Tarlatamab/AMG757	BiTE-Fc	5.7 d	10%	solid tumor	CD3 × DLL	FDA-approved	FDA-approved	188	36.10%	4.20%	71.80%	[109,304]
Tebentafusp	ImmTAC	7.5 h	29–33%	solid tumor	CD3 × gp100 (HLA-A*02:01)	FDA-approved	FDA-approved	505	8%	0%	59%	[50,132,134,305]
Tebentafusp + durvalumab and/or tremelimumab	ImmTAC	N/A	N/A	solid tumor	CD3 × gp100 (HLA-A*02:01)	phase 1b completed	ongoing clinical development	72	14%	0%%	40%	[306]
AMG420/BI836909	BiTE	“short”	0%	relapsed/refractory multiple myeloma	CD3 × BCMA	phase 1 completed	discontinued; potentially due to toxicity and need for infusion	42	31%	12%	48%	[102,115,307]
Flotetuzumab/MGD006	DART	N/A	0.90%	acute myeloid leukemia	CD3 × CD123	phase 1/2 completed	discontinued; replaced by PK-enhanced analog MGD024	88	36%	11.7%%	N/A	[105,107]
AMG330	BiTE	5.49 h	4%	hematological malignancy	CD3 × CD33	phase 1/2 completed	discontinued; toxicity and limited efficacy	35	14.20%	5.70%	66%	[101,104,308,309]
HPN328	TriTAC	71 h	N/A	relapsed/refractory metastatic SCLC, neuroendocrine prostate cancer, and other NEC	CD3 × DLL3 × albumin	phase 1/2 ongoing with updates	ongoing clinical development	9 and 24	44%; 50%	11.1% 0%	≤10%	[46,310]
pasotuxizumab/BAY 2010122/AMG212	BiTE	2–3 h	100%	metastatic castration-resistant prostate cancer	CD3 × PSMA	phase 1 completed	ongoing clinical development	16	25%	6.25%	≤81%	[116]
AMG211/MT111/MEDI-565	BiTE	3.3 h	0% cIV; 96.7% SC	relapsed/refractory gastrointestinal (GI) adenocarcinoma	CD3 × CEA	phase 1 completed	discontinued; high immunogenicity and unable to establish therapeutic window	32	N/A	N/A	16% (all AE)	[24,101,117,311,312]
Etevritamab/AMG596	BiTE	N/A	N/A	EGFRvIII^+^ recurrent glioblastoma (RGBM)	CD3 × EGFRvIII	phase 1 completed	Terminated; business decision	14	12.50%	0%	50%	[118,313]
solitomab/AMG110/MT110	BiTE	4.5 h	11%	refractory solid tumors	CD3 × EpCAM	phase 1 completed	discontinued; on-target dose-limiting toxicity	63	1.5%: 27.7% SD	0%	95%	[112,128]
IMC-C103C	ImmTAC	N/A	N/A	Post = checkpoint cutaneous melanoma	CD3 × MAGE-A4 (HLA)	phase 1 completed	ongoing clinical development	46	61% for PR + SD	0%	31%	[146,147]
IMCnyeso	ImmTAC	25 h	7%	Advanced solid tumor	CD3 × NY-ESO-1 (HLA)	phase 1 completed	ongoing clinical development	27	0%	0%	32%	[314]
PF-06671008	DART-Fc	26.6–45.8 h	3.70%	advanced solid tumors	P- cadherin × CD3	phase 1 completed	discontinued; lack of efficacy, high incidence of AE, may due to unsilenced Fc	27	0%	0%	62.90%	[34,35]
NM21-1480	scMATCH3 (scFv3)	N/A	N/A	unresectable solid tumor	PD-L1 × 4-1BB × albumin	phase 1 completed	ongoing clinical development	26	57% for PR + SD	N/A	0%	[149]
NK cell engager (oncology)												
AFM28	TandAb-Fc	N/A	N/A	relapsed/refractory acute malignant leukemia (R/R AML)	CD16A × CD123	phase 1 completed	ongoing clinical development	12	38.8%	30%	13%	[40]
AFM24 (with atezolizumab)	tetraspecifics	3.24–11.3 h	N/A	WT-EGFR^+^ metastatic NSCLC	CD16A × EGFR	phase 2 completed	ongoing clinical development	15	27%	6.70%	“no new or unexpected toxicity compared to each single agent”	[208,315]
AFM13 (with penbrolizumab)	TandAb	20.6 h	56.60%	relapsed/refractory Hodgkin lymphoma	CD16A × CD30; PD-1	phase 1b completed	ongoing clinical development	30	83%	N/A	7%	[43]
GTB-3550	TriKE	“short as predicted”	N/A	Relapsed/refractory AML and MDS	CD16 × IL-15 × CD33	phase 1 completed	discontinued; replaced by VHH-based GTB-3650	4	0%; 66% for SD	0%	“no signs of clinical immune activation or SAE’s”	[77,78,79,216]
DF1001	TriNKET	N/A	N/A	advanced solid tumors	CD16 × NKp46 × HER2	phase 1/2 completed	ongoing clinical development	36	13.90%	0%	14%	[63]
SAR443579/IPH6101	ANKET	N/A	N/A	Hematological malignancies	CD123 × NKp46 × CD16	phase 1/2 ongoing	ongoing clinical development	59	N/A	“5 CR @ 1 mg/kg”	N/A	[64]
Myeloid cell engager and B cell engager (oncology)												
MP0317	DARPin	70.5 h (1 mg/kg)	N/A	oncology	CD40 × FAP	phase 1 completed	ongoing clinical development	46	N/A	N/A	2.30%	[241,242]
MP0250	DARPin	15–16 day	47.6%	oncology	VEGF × HGF × albumin	phase 1b/2 completed	ongoing clinical development	33	32.1%	0%	51.6%	[72,73]
BDC-1001 (w/wo nivolumab)	ISAC	4.8 d	6.30%	solid tumor	TLR7/8 × HER2	phase 1/2 completed	discontinued; lack of efficacy	118	4.2%PR + 8.5%SD	0%	1.70%	[232,250,316]
NJH395	ISAC	N/A	100%	oncology	TLR7 × HER2	phase 1 completed	discontinued; lack of efficacy	18	N/A	N/A	N/A	[234,235,251]
IMB071703	Xfab/f(ab)2	2.45 h	N/A	recurrent or metastatic advanced solid tumor	CD40 × 4-1BB	phase 1a completed	ongoing clinical development	7	0%; 16.7% SD	N/A	0%	[239]
Immune checkpoint blocker and Immune checkpoint agonist (oncology)												
tebotelimab/MGD013 (w/wo margetuximab)	DART-Fc	11 d	17%	oncology	PD-1 × LAG-3; HER2	phase 1 completed	ongoing clinical development	353	11% (evaluable)	0%	22%	[36,37]
lorigerlimab/MGD019	DART-Fc	12 d	N/A	oncology	PD-L1 × CTLA-4	phase 1 completed	ongoing clinical development	127	25.7	0%	32.30%	[38,39]
erfonrilimab/KN046 (w nab-paclitaxel)	(VHH)4-Fc	111.0–137.4 h	N/A	metastatic triple-negative breast cancer	PD-L1 × CTLA-4	phase 2 completed	ongoing clinical development	27	44%	0%	66.7% (TEAE); 33.3% (SAE)	[293]
erfonrilimab/KN046 (w lenvatinib)	(VHH)4-Fc	N/A	N/A	hepatocellular carcinoma	PD-L1 × CTLA-4	phase 2 completed	ongoing clinical development	55	52%	0%	27.3% TEAE	[295]
erfonrilimab/KN046 (w nab-paclitaxel)	(VHH)4-Fc	N/A	N/A	pancreatic cancer	PD-L1 × CTLA-4	phase 2 completed	ongoing clinical development	53	45.20%	0%	14.3% TRAE	[297]
erfonrilimab/KN046 (w gemcitabine)	(VHH)4-Fc	N/A	N/A	nasopharyngeal carcinoma	PD-L1 × CTLA-4	phase 1 completed	ongoing clinical development	59	15.40%	0%	14% TRAE	[292]
erfonrilimab/KN046 (w chemotherapy)	(VHH)4-Fc	N/A	67.90%	NSCLC	PD-L1 × CTLA-5	phase 2 completed	ongoing clinical development	87	46.00%	0%	72.4% TEAE; 37.9% s-TEAE	[294]
